# Transposable elements and homotypic niches drive immune dynamics and resistance in melanoma epigenetic-based immunotherapy

**DOI:** 10.1126/sciadv.aed1318

**Published:** 2026-08-07

**Authors:** Erika Ciervo, Francesco Ceccarelli, Anna Maria Di Giacomo, Piera Grisolia, Alessia Covre, Zein Mersini Besharat, Antonio De Falco, Francesca Pia Caruso, Luigi Laezza, Luigi Ferraro, Gloria Mas Martin, Daniel Bilbao, Sion Williams, Benjamin Currall, Maria Fortunata Lofiego, Tommaso Sani, Elisabetta Ferretti, Yan Guo, Sean B. Holden, Ines Simeone, Roberta Mortarini, Andrea Anichini, Michele Maio, Teresa Maria Rosaria Noviello, Michele Ceccarelli

**Affiliations:** ^1^ Sylvester Comprehensive Cancer Center, Miller School of Medicine, University of Miami, Miami, FL, USA.; ^2^ Biogem Institute of Molecular Biology and Genetics, Ariano Irpino, Italy.; ^3^ Department of Computer Science and Technology, University of Cambridge, Cambridge, UK.; ^4^ University of Siena, Italy, and Center for Immuno-Oncology, Department of Oncology, University Hospital of Siena, Siena, Italy.; ^5^ NIBIT Foundation Onlus, Siena, Italy.; ^6^ Department of Experimental Medicine, Sapienza University, Rome, Italy.; ^7^ Department of Public Health Sciences, Miller School of Medicine, University of Miami, Miami, FL, USA.; ^8^ Department of Electrical Engineering and Information Technologies, University of Naples Federico II, Naples, Italy.; ^9^ Department of Experimental Oncology, Fondazione IRCCS Istituto Nazionale dei Tumori, Milan, Italy.

## Abstract

Melanoma plasticity drives immune evasion and therapy resistance through dynamic cell-state transitions beyond genetic alterations. Although epigenetic remodeling is central to this process, its impact under therapeutic pressure remains unclear. We profiled longitudinal biopsies from patients with melanoma treated in the phase 1b NIBIT-M4 epi-immunotherapy trial [NCT02608437, DNA (cytosine-5)-methyltransferase 1 inhibitor plus anti–CTLA-4] using single-cell multiome and spatial transcriptomics. Seven malignant meta-programs were identified, including a rare Wnt/β-Catenin melanocytic state and a dedifferentiated neural crest–like state enriched in nonresponders. Spatial analyses showed that homotypic clustering stabilizes resistant programs, with neural crest–like cells forming compact niches. Responders displayed enrichment of antigen presentation/interferon program and coordinated T and B cell expansion, whereas nonresponders retained stable neural crest–like clusters. Epigenetic therapy reactivated transposable elements, priming innate immunity and enhancing immunogenicity. Nuclear factor of activated T cells, cytoplasmic 2 (NFATC2) emerged as a master regulator of neural crest–like states and resistance; its perturbation promoted differentiation and immunogenicity. These findings define mechanisms of resistance and nominate β-Catenin and NFATC2 as therapeutic vulnerabilities.

## Teaser

Epigenetic therapy reactivates transposable elements, reshaping tumor niches and immune responses, driving resistance or response.

## INTRODUCTION

Melanoma is characterized by high heterogeneity and pronounced phenotype switching between cellular states (*1*). This plasticity is driven by nongenetic reprogramming that regulates reversible transcriptional changes (*2*), oscillating between multiple differentiation states that resemble different stages of embryonic development (*3*). For example, dedifferentiated melanoma cells express programs resembling neural crest embryonic developmental stages associated with resistance to immune therapies (*4*). Similarly, tumor-intrinsic programs associated with major histocompatibility complex (MHC) class I down-regulation and MITF^low^/AXL^high^ are hallmarks of resistance to immune checkpoint inhibitors (ICIs) and dedifferentiated phenotypes (*5*). The mechanisms by which epigenetic reprogramming affects specific melanoma cellular states and how they influence therapy response are poorly understood, also due to the lack of suitable human patient data/clinical data (*6*). Recent studies have demonstrated that reprogramming with epigenetic drugs can alter the immune microenvironment and affect melanoma cancer cells (*7*, *8*), as well as enhance the response to immune therapies (*9*, *10*). The main biological activity of epigenetic drugs, such as DNA (cytosine-5)-methyltransferase 1 (DNMT1) inhibitors, is the promotion of the expression of new antigens and activation of master factors belonging to innate immunity pathways, including type I to III interferon (IFN), NFkB, and *TLR10*, corroborating the notion that the rescue of adaptive immunity by ICI may cooperate with the promotion of innate immunity by the epigenetic reprogramming, thus potentially explaining the clinical activity of the combination therapy (*11*). Using bulk gene expression, methylation, and DNA sequencing, we have recently reported that epigenetic immune reprogramming, achieved by DNMT1 inhibition in combination with immune checkpoint inhibition, promotes the up-regulation of endogenous retroviral elements and IFN-stimulated genes (*10*). We also demonstrated that tumor-intrinsic factors associated with genetic immunoediting, coupled with intratumoral immune features, are an efficient predictor of response to both combinatorial therapy and immune checkpoint inhibition. However, bulk analysis lacks cell-type resolution, whereas integrating single-cell technologies would enable dissection of the precise cellular subsets responding to epigenetic and immune-modulatory pressure. Previous studies using single-cell RNA sequencing have revealed distinct transcriptional programs in melanoma cells, including MITF^high^ melanocytic and AXL^high^ invasive states, associated with differential drug sensitivity and plasticity (*12*). Neural crest–like and stress-related tumor-specific programs characterize minimal residual disease, highlighting the dynamic nature of melanoma cell state transitions (*13*). Other cellular states associated with antigen-presenting and undifferentiated phenotypes were also uncovered with single-cell technologies (*1*). Pozniak *et al.* (*14*) recently integrated multimodal single-cell and spatial transcriptomic data from patients with melanoma treated with ICIs, revealing how transitions between melanoma cell states, particularly the shift from melanocytic to mesenchymal-like phenotypes, are associated with immune evasion and therapeutic resistance. The lack of genetic alterations supporting the recurrent melanoma cellular states observed in these studies highlights the role of nongenetic mechanisms in influencing phenotypic switching between tumor cellular states in melanoma, as well as their cross-talk with the microenvironment and association with response to treatment. To uncover rare or transitional cellular phenotypes, lineage-specific transcriptional rewiring, and spatially confined tumor-immune network, ultimately refining the evolution of cellular states under immune therapy and elucidating resistance mechanisms not apparent at the bulk level, we have generated single-cell multiome coupled with high-resolution spatial transcriptomics of longitudinal biopsies from patients with melanoma treated in the phase 1b NIBIT-M4 trial, with the association of the anti–CTLA-4 ipilimumab, and the DNMT1 guadecitabine (*9*, *10*). Here, we show how epi-immunotherapy reshapes melanoma ecosystems. We demonstrate that clinical response/benefit is associated with progressive enrichment of an *Antigen presentation/Interferon* program, whereas resistance is marked by the persistence of a *Neural crest–like* state. Consistent with recent evidence that homotypic clustering stabilizes glioblastoma states and constrains transcriptional heterogeneity (*15*), we observed that resistant neural crest–like programs form compact homotypic niches enriched in nonresponders. Our data extend this principle by showing that different melanoma states adopt distinct spatial strategies: While *Neural crest–like* programs rely on cohesive niches for stability, *Wnt/*β*-Catenin* cells can exist in both clustered and dispersed configurations, with dispersion linked to higher plasticity. Moreover, we show that transposable element (TE) reactivation driven by epigenetic-based immunotherapy contributes both to regulatory and antigenic signals, highlighting a dual role in shaping tumor-immune dynamics. Last, we identify nuclear factor of activated T cells, cytoplasmic 2 (NFATC2)–driven plasticity as a central therapeutic vulnerability, whose inhibition may promote redifferentiation and overcome the resistant *Neural crest*–*like* state.

## RESULTS

### Longitudinal single-cell multiome profiling of the NIBIT-M4 clinical trial

**Fig. 1.**
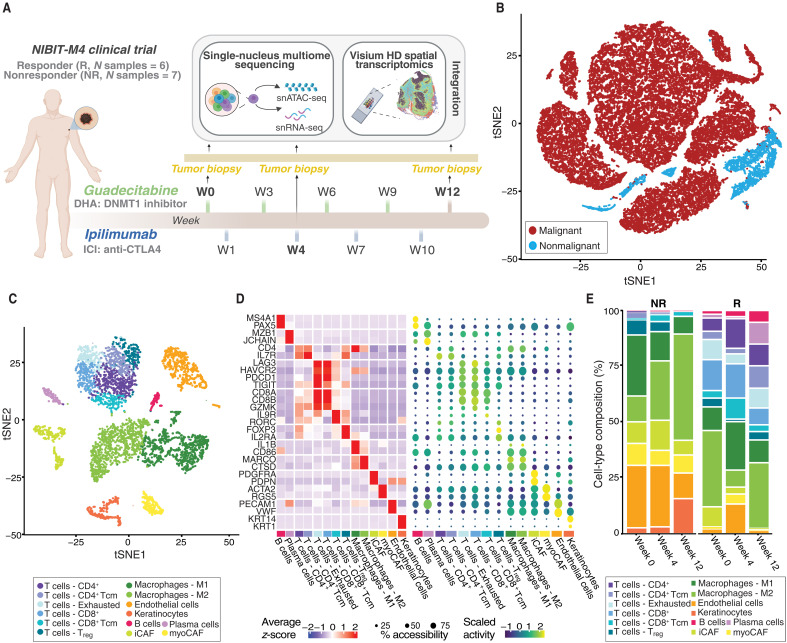
Multiomic characterization of the NIBIT-M4 cohort. (**A**) Schematic overview of the study design. Tumor biopsies were collected from patients classified as responders (R, *n* = 6) and nonresponders (NR, *n* = 7) at baseline (W0), week 4 (W4), and week 12 (W12), and profiled using single-nucleus Multiome sequencing (snRNA-seq and snATAC-seq) along with Visium HD Spatial Transcriptomics. (**B**) t-Distributed stochastic neighbor embedding (t-SNE) of multimodal snRNA-seq and snATAC-seq data colored by malignant and nonmalignant cell populations. (**C**) t-SNE of the tumor microenvironment (TME) using snRNA-seq data, colored by cell types. Abbreviations: Tcm, T central memory; T_reg_, regulatory T cell; iCAF, inflammatory cancer-associated fibroblasts; myoCAF, myofibroblastic cancer-associated fibroblasts. (**D**) Expression and activity of marker genes across cell types shown as average *z*-score expression (left) and scaled gene activity (dot plot, right). (**E**) Bar plot showing TME cell-type composition in NR and R patients across time points. Part of (A): Created in BioRender. T. M. R. Noviello (2026) https://BioRender.com/uu7fh9q.

To map the dynamic evolution of distinct malignant cell subpopulations and the tumor microenvironment (TME) over the course of epigenetic therapy in melanoma, we performed longitudinal profiling using the 10x Genomics Chromium Single Cell Multiome platform on tumor biopsies collected at baseline (week 0) and during treatment (weeks 4 and 12) from patients with melanoma treated in the NIBIT-M4 trial (Fig. 1A). We generated paired single-nucleus RNA sequencing (snRNA-seq) and ATAC-seq (snATAC-seq) libraries across 13 tumor specimens obtained from five patients, two responder (R) and three nonresponder (NR) patients. Following integrated quality control (QC) across both modalities, we retained 38,029 high-quality cells with matched transcriptomic and chromatin accessibility profiles, comprising 58,670 cells that passed RNA filters and 55,850 cells that passed ATAC filters.

Cells were classified as malignant (*n* = 34,822) based on inferred copy number alterations (CNAs) from gene expression data (*16*) (fig. S1A). Inferred copy number profiles were highly concordant with those obtained from the bulk exome sequencing samples with matched normal previously reported (*10*) (table S1). Unsupervised dimensionality reduction, integrating both transcriptomic and chromatin accessibility modalities, consistently separated nonmalignant from malignant compartments, as visualized using t-distributed stochastic neighbor embedding (t-SNE) (Fig. 1B). Malignant cells were largely intermixed between time points, indicating that global transcriptional programs were conserved across the study period. Nonetheless, specific clusters appeared enriched for cells collected at week 4, reflecting the abundance of cells collected at week 4 (fig. S1B).

To characterize changes in the TME during treatment, nonmalignant cell types (*n* = 4968) were identified through clustering analysis followed by cell-type annotation (Fig. 1C and table S2). Given the limited availability of cell-type–specific chromatin accessibility markers, annotation was performed using the snRNA-seq modality, leveraging validated reference gene signatures and canonical marker gene expression (Fig. 1D, left). We identified multiple T cell subpopulations, including CD8^+^, CD4^+^, exhausted, regulatory (T_reg_), and central memory (Tcm) T cells, as well as B cells and plasma cells. Macrophages were further classified into M1 and M2 polarized states, while the stromal compartment comprised cancer-associated fibroblasts (CAFs), distinguished into inflammatory CAFs (iCAFs) and myofibroblastic CAFs (myoCAFs) (*17*). In addition, endothelial cells and keratinocytes were also detected. The snATAC-seq data corroborated the reliability of TME cell-type annotation derived from transcriptomic data. The same marker genes that exhibited cell-type–specific expression in the RNA modality also showed consistent patterns of chromatin accessibility in the corresponding TME clusters, as measured by gene accessibility scores (Fig. 1D, right).

As a longitudinal/prospective clinical trial, this study enabled the longitudinal tracking of temporal dynamics within the tumor ecosystem at single-cell resolution. Combination therapy induced a progressive immune engagement and functional remodeling of CD4^+^ and CD8^+^ T cells, as indicated by the transcriptional and epigenetic activation of the PD-1 signaling pathway (fig. S1C), indicative of a sustained and coordinated immune engagement over time and consistent with previous findings from bulk analyses (*10*). We further observed that the composition of TME cell populations varied over time within response groups (Fig. 1E). In NR patients, the TME remained largely immunosuppressive throughout treatment, characterized by a dominance of M2-polarized macrophages, limited T cell infiltration, and persistent stromal elements such as iCAFs and keratinocytes, reflecting poor immune activation. In contrast, R patients exhibited dynamic TME remodeling. By week 4, there was a notable increase in CD8^+^ and CD4^+^ T cells, exhausted T cells, and M1 macrophages, indicating early immune activation. This trend further intensified by week 12, with a marked expansion of T cells accompanied by the emergence of B cells and plasma cells, consistent with a coordinated and sustained antitumor immune response. Consistently, expression levels of key immune checkpoint genes were significantly elevated in T cells from R patients over time (fig. S1D), reinforcing the sustained immune activation and adaptive feedback typical of effective immune responses.

Overall, our longitudinal single-cell analysis confirms that effective response to epi-immunotherapy in melanoma, combining DNMT1 inhibition with ICI, is associated with dynamic and coordinated remodeling of the TME, characterized by progressive immune activation and diversification over the course of treatment.

### Dissecting the heterogeneity of melanoma cell states under epi-immunotherapy

**Fig. 2.**
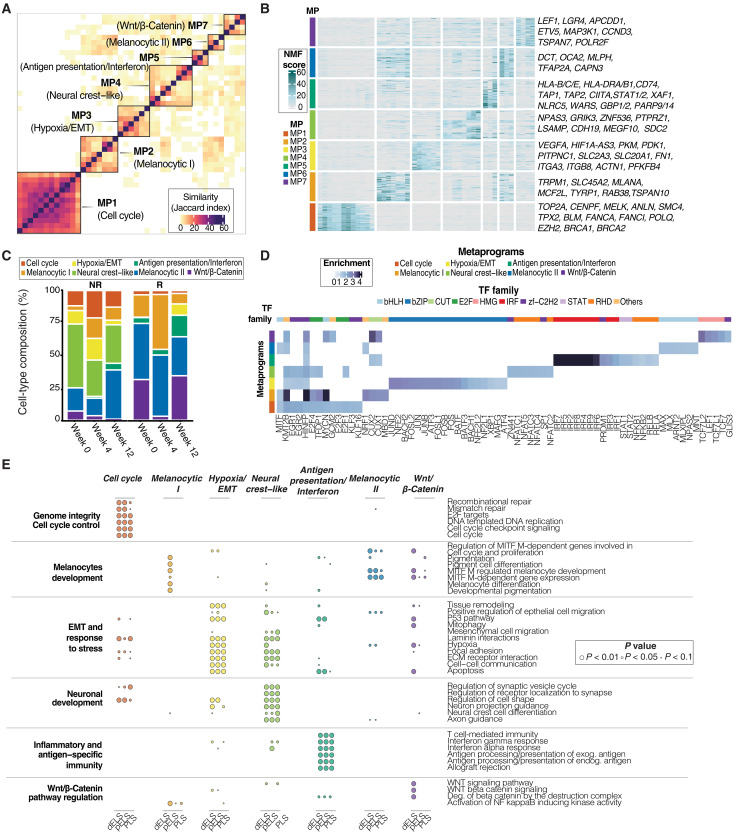
Characterization of metaprograms and their regulatory activity. (**A**) Similarity matrix of NMF programs based on gene overlap (Jaccard index). Programs are clustered and grouped into metaprograms (MPs), each annotated with a functional description derived from the top 50 genes. (**B**) Heatmap showing NMF scores of genes within MP signatures. Rows represent genes, and columns represent programs composing each MP. Selected genes for each MP are highlighted. (**C**) Bar plot of MP composition in NR and R patients across time points. (**D**) Motif enrichment map of TF binding motifs (columns) across MPs (rows), highlighting candidate regulators of transcriptional states. TFs are color coded according to their families. (**E**) Bubble plot of pathway enrichment for MP-specific TF target genes identified through cCRE-TF-gene linkage analysis. Bubble size indicates enrichment significance (*P* value), and color denotes the corresponding MP.

To resolve the complexity of melanoma under epi-immunotherapy, we applied a non–negative matrix factorization (NMF) framework to snRNA-seq data from malignant cells, enabling the identification of shared transcriptional programs (*18*). This analysis revealed seven consensus meta-programs (MPs), representing distinct cellular identities or activity states that were conserved across tumors. Each of the seven NMF-derived MPs exhibited unique gene expression patterns, as well as strong internal correlation and gene overlaps (Fig. 2A and fig. S2A). Subsequently, we assigned each malignant cell to a specific MP based on the highest MP signature score (see Materials and Methods) (fig. S2B). Cells falling below a simplicity score threshold of 0.1, and therefore not confidently classified, were deemed undefined and excluded from subsequent analyses.

Taking advantage of the joint multiomic resolution of our tumor specimens under epigenetic therapy, we first assessed the extent to which transcriptional states from snRNA-seq are reflected in chromatin accessibility profiles from snATAC-seq, quantified as the percentage of cells with correctly predicted annotations via label transfer across the two modalities (fig. S2C). The extent to which accessibility profiles predict transcriptional states can serve as a measure of epigenetic plasticity, where cells with uncertain predictions are indicative of more plastic states (*19*). Malignant cells exhibited greater prediction variability than nonmalignant cells in the TME, with chromatin accessibility often compatible with more than one transcriptional state. The degree of such flexibility varied across tumor cell states: While some MPs retained stable, concordant identities across modalities, others exhibited greater heterogeneity. In particular, MP1 was characterized by the highest plasticity. These findings are in line with recent work in other tumor types (*19*), supporting the notion that tumor cell states span a continuum shaped by chromatin remodeling, transitioning between states.

Then, to interpret the biological relevance of each MP, we analyzed the top 50 genes ranked by NMF scores for each MP (Fig. 2B and table S3) and assessed their functional enrichment to elucidate underlying biological processes (table S4). When interpretation was challenging, we also analyzed the differential expression profile of each MP compared to the others (table S5), followed by functional enrichment analysis (see Materials and Methods and table S6).

MP1 was characterized by genes involved in cell proliferation and DNA repair mechanisms, highlighting biological processes such as cell cycle regulation, chromosomal stability, DNA damage response, and mitosis. It expresses key genes such as *TOP2A*, *MELK*, and *ANLN*, which regulate mitotic progression, spindle assembly, and cytokinesis, hallmarks of rapidly dividing melanoma cells (*20*–*22*). The enrichment of DNA repair factors, including *BRCA1*, *BRCA2*, *FANCA*, *FANCI*, *POLQ*, and *BLM*, suggests that melanoma cells in this program rely heavily on homologous recombination and replication stress response pathways to preserve genomic integrity under proliferative stress (*23*). This metaprogram, hereafter referred to as *Cell cycle*, recapitulates a proliferative transcriptional phenotype, consistently identified in human tumor biopsies, including melanoma (*3*, *12*, *14*, *18*) (fig. S2D).

MP2, hereafter referred to as *Melanocytic I*, was defined by a melanocytic-like differentiated phenotype, characterized by the expression of genes such as *TRPM1*, *SLC45A2*, *MLANA*, *TYRP1*, and *RAB38*, which are involved in melanin biosynthesis, vesicle trafficking, and melanocyte differentiation (*24*–*27*). Furthermore, pathways regulated by *MITF* were significantly enriched among the top-ranked gene sets (table S3), underscoring the central role of MITF-driven transcriptional programs in defining this metaprogram. This is consistent with the well-established melanocytic differentiation axis, which is linked to MITF activity and a more proliferative cellular state (*2*, *3*, *12*, *14*, *18*, *28*) (fig. S2D).

MP3 was associated with cellular responses to hypoxia and stress, and was thus designated as *Hypoxia/EMT*. Key genes such as *VEGFA*, *HIF1A-AS3*, and *PKM* facilitate angiogenesis and regulate hypoxia-inducible factor signaling, enabling adaptation to low oxygen levels (*29*–*31*). Moreover, a metabolic stress phenotype was evident through the expression of *PDK1***,** *PFKFB4*, *PITPNC1, SLC2A3*, and *SLC20A1* (*32*–*34*). In addition, genes such as *ITGA3*, *ITGB8*, *FN1*, and *ACTN1* mediate cell adhesion and extracellular matrix (ECM) remodeling (*35*), further supporting the invasive and hypoxia-adapted phenotype of these melanoma cells.

MP4 was characterized by the presence of genes involved in neural signaling pathways, including *GRIK3*, a neurotransmission receptor subunit; *ZNF536*, a brain-specific zinc finger protein; *LSAMP*, a neural adhesion molecule; *MEGF10*, a phagocytic receptor highly expressed in glial cells; and *CDH19*, a cadherin contributing to neural adhesion. These features suggest that this metaprogram reflects a dedifferentiated state of melanoma cells, transitioning toward a neural crest–like phenotype (*2*, *3*, *12*, *14*, *18*, *28*, *36*–*38*) (fig. S2D), and was therefore named *Neural crest–like*. This MP was enriched exclusively in NR patients (Wilcoxon test, *P* value = 0.02; Fig. 2C), as expected for an invasive phenotype, previously associated with resistance to ICI (*39*). In addition, the expression of *PTPRZ1* and *SDC2* further supports the acquisition of stem cell–like traits and therapy resistance, potentially mediated by interactions with immunosuppressive cells in the TME. To further confirm this point, we quantified stemness at the single-cell level using the top 100 genes of the stemness signature described by Malta *et al.* (*40*). MP4 showed the highest enrichment for stemness among all identified cellular states (fig. S2E). The dedifferentiation features of the identified metaprograms were also evaluated using two additional orthogonal approaches: the IFN-γ–induced dedifferentiation signature from (*41*) and a minimal marker-based dedifferentiation index defined as the ratio of *NGFR* expression to the average of *PMEL* and *MLANA* expression (*42*, *43*). Both approaches showed that the MP3, MP4, and MP5 states exhibit the highest dedifferentiation scores compared to other programs (fig. S2E). Last, cell-cell interaction analysis in NR patients revealed activation of the pro-tumorigenic Pleiotrophin signaling pathway (*44*), involving these genes and suggesting a cross-talk between this cell state and CAFs (fig. S2F).

MP5 exhibits remarkable immune-related traits, with genes involved in the antigen presentation machinery, both MHC I and II, including *HLA-B*, *HLA-C*, *HLA-E*, *HLA-DRA*, *HLA-DRB1*, *CD74*, *TAP1*, *TAP2*, and *CIITA*. In addition, transcription factors (TFs) and immune effectors such as *STAT1*, *STAT2*, *NLRC5*, *XAF1*, *WARS*, *GBP1*, *GBP2*, *PARP9*, and *PARP14*, as well as a suite of interferon-stimulated genes, collectively justify its designation as the *Antigen presentation/Interferon* metaprogram. Consistent with previous findings (*14*, *18*), these tumor cells exhibited an immunologically active phenotype within the TME, contributing to improved response. Accordingly, MP5 was more enriched in R versus NR patients (Wilcoxon test, *P* value = 0.07; Fig. 2C). Cell-cell interaction analysis in R patients further revealed robust activation of both MHC class I and, notably, class II pathways (fig. S2G). Cells in this program displayed a progressive up-regulation of immunoproteasome subunits (*PSMB8*, *PSMB9*, and *PSMB10*) across treatment time points (fig. S2H), whereas constitutive proteasome components (*PSMB5*, *PSMB6*, and *PSMB7*) showed no distinct pattern. This suggests that therapy induces an immunoproteasome-dependent antigen processing machinery, which has been previously associated with a favorable ICI response in melanoma (*45*). MP5 was also characterized, together with MP4, by a higher level of stemness features than other MPs (fig. S2E).

MP6 was also associated with a melanocytic-like phenotype, highlighted by the expression of key pigmentation-related and MITF-dependent genes (table S4), including *DCT*, *OCA2*, *MLPH*, *TFAP2A,* and *CAPN3* (*46*–*50*). A strong correlation and substantial gene overlap further corroborated this phenotypic link with the previously defined *Melanocytic I* (Fig. 2B and fig. S2A), as well as with other known melanocytic cell states (fig. S2D). On the basis of this evidence, we designated MP6 as *Melanocytic II.*

MP7 was marked by strong activation of the Wnt/β-Catenin signaling pathway, evidenced by high expression of *LEF1*, a key TF downstream of β-Catenin. *LEF1* is known to play a central role in melanocytic differentiation, as Wnt/β-Catenin signaling is critical for the early specification and maturation of melanocyte precursors from the neural crest (*51*, *52*). Further support for Wnt pathway activation came from its selective up-regulation in MP7 compared to other melanocytic programs (Melanocytic I/II), along with the highest expression of the *CTNNB1* (β-Catenin) gene (fig. S2I). On the basis of these findings, we classified MP7 as a distinct melanocytic-like metaprogram driven by such signaling and accordingly named it *Wnt/*β*-Catenin*.

Together, these results reveal a heterogeneous landscape of malignant cell states, each defined by distinct transcriptional and functional traits, that may collectively influence the balance between therapeutic sensitivity and resistance in melanoma under epi-immunotherapy.

### Integrated analysis of epigenetic regulation and TF activity in melanoma cell states under epi-immunotherapy

The availability of paired RNA and ATAC measurements enabled not only the characterization of dynamic transcriptional states and DNA regulatory element activities but also facilitated direct mapping of epigenetic gene regulation to gene expression changes.

To investigate the regulatory mechanisms driving melanoma cell metaprograms and their changes under therapy, we performed TF motif enrichment analysis on differentially accessible (DA) chromatin regions identified from snATAC-seq data. Across metaprograms, we identified an average of 19,787 accessible peaks with significant enrichment (average log_2_FC ≥ 0.58 and adjusted *P* value < 0.05). To identify key TFs controlling each metaprogram, enriched motifs within these peaks were examined. We prioritized motifs with (i) fold enrichment ≥1.3, (ii) retaining the most enriched motif when multiple motifs correspond to the same TF, and (iii) compiling the top 30 most enriched TF motifs per metaprogram. A total of 113 unique TFs were identified across the seven MPs (table S7). From these, a subset of 69 MP-specific TFs was visualized to highlight the transcriptional programs and phenotypic identities of the MPs, as previously defined by snRNA-seq (Fig. 2D).

*MITF* was confirmed as the principal regulator of the melanocytic MPs, in line with its well-established role in melanocyte differentiation and pigmentation. The *Hypoxia/EMT* state showed strong enrichment for TFs from the bZIP (basic leucine zipper) family, which are known mediators of stress responses, cellular adaptation, and epithelial-to-mesenchymal transition, as *ATF3* and the FOS/JUN (AP-1) complex and *BACH1* (*53*, *54*). Similarly, the *Antigen presentation/Interferon* metaprogram was marked by specific and strong enrichment of IRF and STAT family TFs, driving an immunologically active phenotype. The *Wnt/*β*-Catenin* metaprogram featured enrichment of HMG-box family TFs, including *LEF1* and *TCF7L2* (*55*), which are well-known regulators of Wnt signaling (fig. S3A). The *Neural crest–like* metaprogram appeared to be predominantly regulated by members of the nuclear factor of activated T cells (NFAT) family, TFs best known for their role in calcium-dependent signaling and involved in the control of T cell development, and T cell differentiation (*56*). As a complementary approach, enhancer-driven gene regulatory networks (eGRNs) were reconstructed by integrative analysis of paired snRNA-seq and snATAC-seq multiome data using the SCENIC+ pipeline. This analysis further supported the involvement of the same key TFs identified via motif enrichment in regulating metaprogram-specific gene expression (fig. S3B, top, and table S8) providing additional support for their regulatory role. To validate the specificity of these TFs within the identified metaprograms, we computed regulon activity scores in two external contexts: the scRNA-seq melanoma cohort of Pozniak *et al.* (*14*) (fig. S3B, middle) and two additional profiled samples not used in the inference of the regulon (fig. S3B, bottom). In both datasets, the regulon activity patterns were broadly consistent with those observed in the discovery cohort, confirming the robustness and generalizability of the inferred metaprogram-specific regulatory networks. Because both additional samples were derived from two responder patients, the *Neural crest*–*like* metaprogram was not represented and consequently the TFs specifically associated with this program could be confounded by similar pathways active in other metaprograms.

To further dissect the contribution of key TFs to MP-specific regulatory phenotypes, we conducted an integrative cis-regulatory element (CRE)–TF-gene linkage analysis. For each TF, motif-enriched peaks overlapping ENCODE-annotated promoter- and enhancer-like elements (both proximal and distal) were retained (*n* = 61,054), with 6.84% mapping to promoter-like signatures (PLS), 17.84% mapping to proximal enhancer-like signatures (pELS), and 75.32% mapping to distal enhancer-like signatures (dELS). Consistently, regulatory elements inferred by SCENIC+ showed strong overlap with ENCODE-annotated enhancer-like signatures, both proximal and distal elements (fig. S4A and table S9), with skin-derived datasets ranking among the highest-overlapping tissues across 49 ENCODE tissue types (table S10), further supporting the structural robustness and the biological relevance of the reconstructed eGRNs. Genes linked to these CREs (*n* = 16,237) were then intersected with MP-specific up-regulated genes identified from differential expression analysis on snRNA-seq data (table S5), leading to the identification of a high-confidence set of 1953 MP-specific target genes of enriched TFs across PLS, pELS, and dELS layers.

Downstream pathway analysis on these genes (Fig. 2E and table S11) revealed that melanocyte development-related pathways were enriched across multiple MPs, consistent with the tumor cell origin and largely independent of the CRE category. However, distinct phenotypes emerged when focusing on dELS, particularly for the *Melanocytic I* and *Wnt/*β*-Catenin* MPs, highlighting the potential regulatory importance of distal elements in defining these cell states. *Neural crest–like* cells showed strong enrichment for neural development pathways and Epithelial-to-Mesenchymal Transition (EMT)–related processes such as focal adhesion and ECM-receptor interaction (*P* value <0.01), reinforcing their neural origin and invasive phenotype. The *Antigen presentation/Interferon* state was highly specific for inflammatory and antigen-specific immunity pathways.

Last, the TFs inferred as drivers of each metaprogram were significantly enriched for direct binding at the corresponding accessible peaks, with many enrichments supported by chromatin immunoprecipitation sequencing (ChIP-seq) experiments performed in melanoma cell lines (fig. S4B and table S12), providing orthogonal experimental confirmation of the inferred regulatory architecture. Collectively, these results indicate that distinct TF regulatory networks, mediated via promoter and enhancer elements, underlie the functional heterogeneity and phenotypic specialization of melanoma cell states during epi-immunotherapy.

### Spatial clustering stabilizes resistant tumor states and modulates phenotypic plasticity

**Fig. 3.**
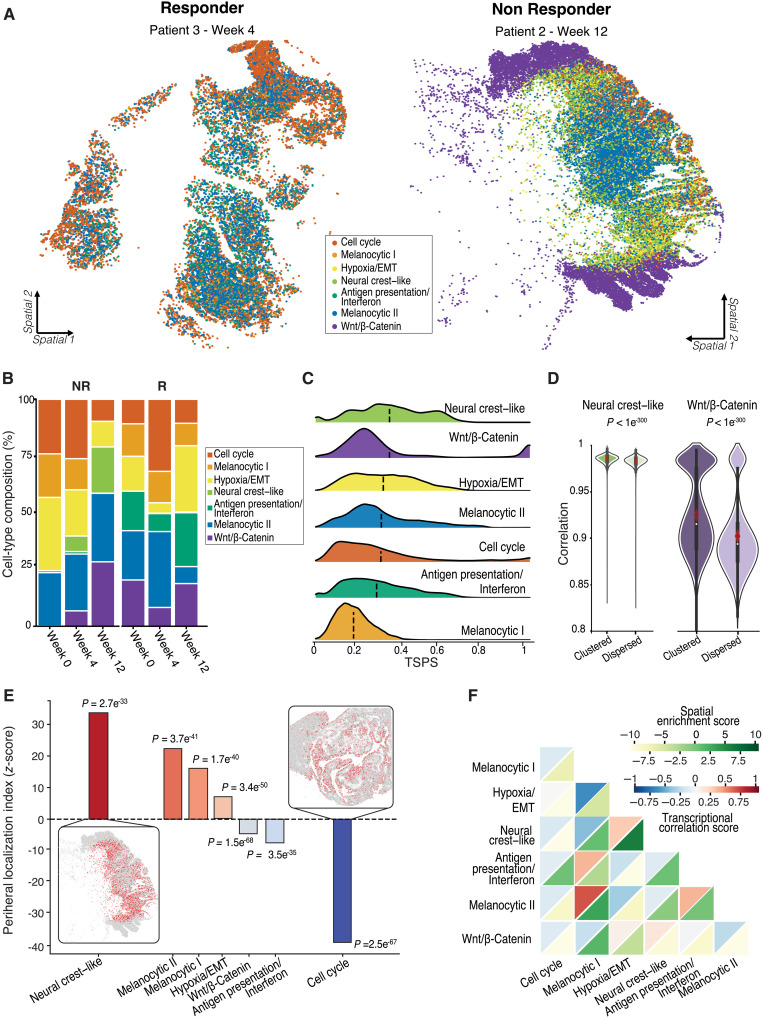
Analysis of the spatial organization of malignant cell states in Visium HD samples. (**A**) Spatial arrangement of metaprograms (MPs) for a responder (R, left) and nonresponder (NR, right) patient. (**B**) Bar plot of MP composition in NR and R patients across time points. (**C**) Tumor State Proximity Score (TSPS) distribution of each MP. (**D**) Pairwise correlations and statistical significance (two-sided Mann-Whitney *U* test *P* value) between the expression profiles of *Neural crest–like* and *Wnt/*β*-Catenin* cells stratified in clustered and dispersed groups based on their TSPS. (**E**) Peripheral Localization Index (PLI) for each metaprogram, measuring the tendency of each cluster to localize near the tissue boundary (empirical permutation test, two-tailed *P* value, 500 randomizations). Spatial maps of the MPs with the highest (*Neural crest–like*) and lowest (*Cell cycle*) PLI are shown to illustrate boundary-enriched versus centrally enriched localization patterns. (**F**) Spatial colocalization patterns (upper triangles) and transcriptional correlations (lower triangles) between MPs.

To analyze how melanoma cells interact with their microenvironment and how cellular neighborhoods shape tumor heterogeneity and progression, we investigated the heterogeneity and spatial organization of metastatic melanoma lesions under therapy in nine samples from four patients (R *n* = 2, and NR *n* = 2) using 10x Genomics VisiumHD profiling. We performed nuclei segmentation on the high-resolution hematoxylin and eosin (H&E) stained images to delineate individual nuclei (see Materials and Methods), resulting in an average of 113,366 cells per sample. QC filters were applied to remove cells with high mitochondrial gene content and low complexity; a total of 736,028 cells passed the QC step, and their gene expression counts were normalized and log_2_ transformed. After dimensionality reduction and graph-based clustering, cells were classified as malignant and nonmalignant based on the expression of well-known melanoma markers (*PMEL*, *TYRP1*, *DCT*, and *MLANA*). On average, 50% of cells were classified as malignant, and 50% were classified as nonmalignant, with fractions of malignant cells and nonmalignant cells ranging from 30 to 65% and 35 to 70%, respectively. To uncover the spatial dynamics of melanoma cellular states, we annotated malignant metaprograms (Fig. 3A) by performing Optimal Transport (OT), based on Fused Gromov-Wasserstein distance coupled with spatial constraints (*57*, *58*), and assigning to each Visium HD cell the cell-type label of the most probable match from the snRNA-seq modality (see Materials and Methods). Visium HD–derived cell-type composition was consistent with snRNA-seq [Pearson correlation *r* = 0.47, 95% confidence interval (CI) (0.24 to 0.64), *P* value = 3.9 × 10^−6^], confirming overall agreement across platforms. In addition, the spatial activity of the identified TFs acting as master regulators of the various metaprograms recapitulated the MP-specific tissue regions identified by our analysis, confirming the robustness of the alignment between the two platforms with OT (fig. S4C).

We first investigated tumor cell–intrinsic functional states in the context of their immediate malignant neighbors (homotypic interactions). Consistent with the RNA-seq modality, we observed a highly significant enrichment of *Neural crest–like* in NRs compared to Rs [Fisher’s exact test *P* value <0.05, odds ratio = 51.7, 95% CI (46.9, 57.1)] (Fig. 3B). Conversely, *Antigen presentation/Interferon* was statistically enriched in Rs compared to NRs [Fisher’s exact test *P* value <0.05, odds ratio = 0.033, 95% CI (0.031, 0.035)]. Spatial self-association of tumor states was captured using a Tumor State Proximity Score (TSPS; radius *r* = 500, see Materials and Methods). Briefly, the TSPS measures the spatial closeness between cells belonging to the same tumor state: Higher values denote spatial clustering and lower values reflect cellular dispersion (Fig. 3C). *Neural crest–like* cells formed the most compact clusters (TSPS mean = 0.35), consistent with their enrichment in NRs and suggesting the existence of a spatially consolidated treatment-resistant niche within such tumors. *Wnt/*β*-Catenin* exhibited a rare bimodal TSPS distribution (TSPS mean = 0.34), indicating that in this transcriptional program, either compact niches or dispersed cell subpopulations can spatially exist. *Antigen presentation/Interferon* showed a broad value distribution with moderate spatial clustering (TSPS mean = 0.28), whereas *Melanocytic I* displayed the most dispersed spatial arrangement (TSPS mean = 0.18). To test whether clustered homotypic interactions support coordinated cellular functions, following an approach similar to Migliozzi *et al.* (*15*), we stratified *Neural crest–like* and *Wnt/*β*-Catenin* cells according to their TSPS distributions (top ≥75th percentile as clustered, bottom ≤25th percentile as dispersed) and computed pairwise transcriptomic correlations within each subgroup. In both programs, clustered cells showed significantly higher pairwise correlations than dispersed cells, indicating that spatial clustering aligns with reduced transcriptional heterogeneity (Fig. 3D).

To assess how spatial context may influence transitions between tumor phenotypes, we applied CellRank (*59*) along pseudotime to compute transition probabilities between spatial configurations of each cell state. Clustered cells were more likely to remain in the same state and maintain their spatial organization, while dispersed cells exhibited higher probabilities of transitioning to alternative states or spatial configurations (fig. S5). Notably, *Antigen presentation/Interferon* and *Hypoxia/EMT* states showed the highest homotypic transition probabilities, highlighting their propensity for strong stabilization through spatial clustering.

Overall, these findings indicate that spatial clustering reinforces cell state identity, whereas dispersion enhances transcriptional plasticity and phenotypic instability.

### Spatial compartmentalization governs functional colocalization of tumor states

We then examined the composition of surrounding nonmalignant cells (heterotypic interactions) to understand how the broader microenvironment shapes tumor cell states. We computed a Peripheral Localization Index (PLI) for each metaprogram, with negative values indicating peripheral enrichment, and positive values reflecting central localization (see Materials and Methods). *Neural crest–like* cells exhibited the highest central localization (PLI *z*-score > 30, *P* value = 2.7 × 10^−33^, Fig. 3E), alongside the highest TSPS among all the MPs, indicating that these cells not only occupy the tumor core but also form densely clustered domains. This co-occurrence of central positioning and spatial compactness corroborated the notion that *Neural crest–like* represents a functionally cohesive, treatment-resistant subpopulation of cells. Other programs, including *Melanocytic I* and *II*, as well as *Hypoxia/EMT,* were enriched toward the tumor center, although to a lesser extent. In contrast, *Cell cycle* was predominantly localized to the tumor periphery, with a markedly negative PLI *z*-score (*P* value = 2.5 × 10^−67^), followed by *Antigen presentation/Interferon* and *Wnt/*β*-Catenin*, which were similarly enriched at the tumor margins, albeit to a lesser extent. We further independently validated these findings using high-resolution Xenium spatial transcriptomic data from (*14*) (fig. S6A), after annotation with OT-derived metaprogram identities (table S13).

We then examined the spatial colocalization and transcriptional correlations between malignant metaprograms as a measure of functional compartmentalization. We observed a positive association between *Melanocytic I* and *Melanocytic II*, indicating that these programs not only share similar melanocytic-like transcriptional identities but also occupy overlapping spatial niches (Fig. 3F). Similar co-occurrence patterns were observed for *Hypoxia/EMT* with *Neural crest–like*, and *Melanocytic II* with *Antigen presentation/Interferon.* In contrast, *Cell cycle* showed strong negative colocalization and transcriptional correlation with *Hypoxia/EMT* and *Wnt/*β*-Catenin*, suggesting spatial segregation of these states within distinct tumor regions.

Collectively, these results indicate that malignant heterogeneity is organized not only by transcriptional divergence but also by spatial compartmentalization, with specific programs preferentially colocalizing to support divergent functional strategies within the tumor ecosystem.

### Spatial coordination of malignant and immune cells underlies response heterogeneity and ecosystem architecture

**Fig. 4.**
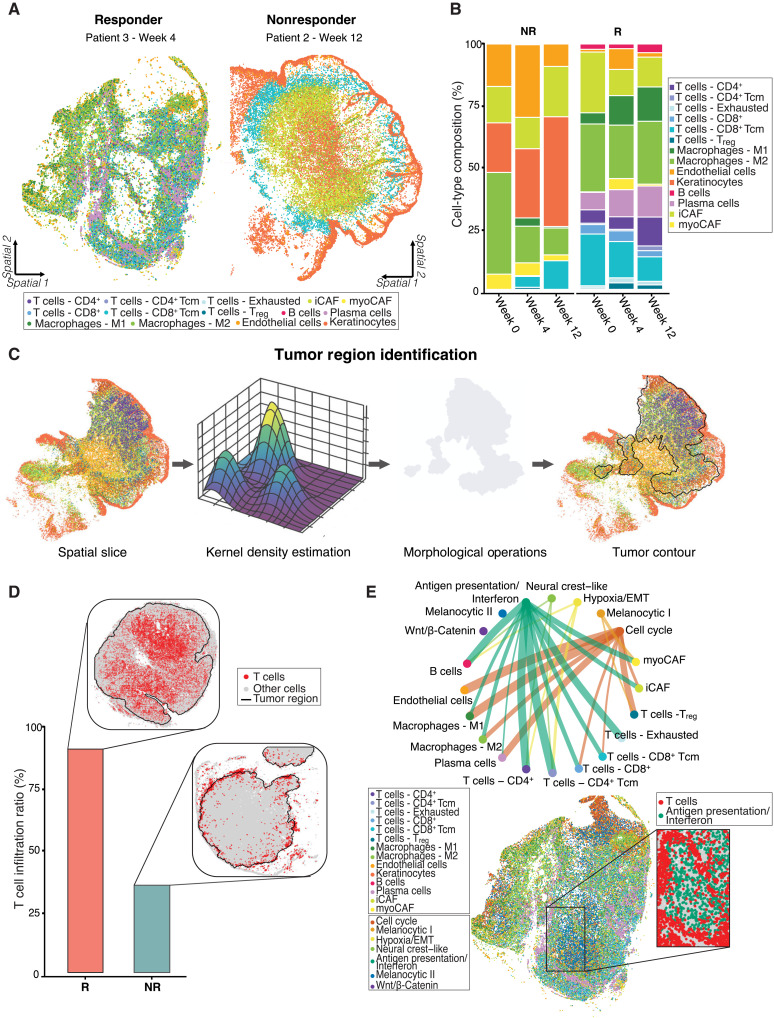
Analysis of the spatial organization of nonmalignant cell states in Visium HD samples. (**A**) Spatial arrangement of nonmalignant cell states for a responder (R, left) and a nonresponder (NR,right) patient. (**B**) Bar plot of nonmalignant composition in NR and R patients across time points. (**C**) Computational pipeline for tumor region identification. Gaussian kernel density estimation (KDE), along with an adaptive binarization threshold, is followed by morphological operations (closing and dilation) to extract a valid tumor contour. (**D**) T cell infiltration ratio (% of T cells inside the tumor region) between NR and R patients. (**E**) Enrichment of malignant metaprograms (MPs) around nonmalignant cell types. Top: MPs-nonmalignant interaction for an R patient, where the width of the edge is proportional to the strength of the interaction. Bottom: spatial map highlighting colocalization of *Antigen presentation/Interferon* and T cells.

To comprehensively map the tumor ecosystem and its interactions with adjacent nonmalignant cells (heterotypic interactions), we first annotated nonmalignant subpopulations using the same OT methodology previously applied to malignant cells (Fig. 4A). Consistent with the RNA-seq modality, the TME of the NR patients remained immunosuppressive throughout treatment, marked by persistent stromal elements and minimal immune infiltration (Fig. 4B). In contrast, R patients exhibited a progressive increase in M1 macrophages, T cells, B cells, and plasma cells, reflecting a spatially active immune engagement and a shift toward antitumor immunity.

Then, tumor contours were delineated for each sample using a kernel density estimation (KDE) and morphological operations (Fig. 4C; see Materials and Methods). Consistent with previous reports (*14*, *18*), we observed a significantly higher T cell infiltration in R compared to NR patients (Fig. 4D). This finding was further corroborated in high-resolution Xenium spatial transcriptomic data (*14*), when considering the relative percentage of lymphocytes within the tumor region (fig. S6B).

To investigate how malignant programs interact with their surrounding microenvironment, we quantified the local neighborhood composition of tumor cells, assessing which malignant states were preferentially enriched near distinct nonmalignant cell types within a defined spatial radius. To distinguish true biological organization from random proximity, observed neighborhoods were compared against a permutation-based null model in which malignant program labels were shuffled while cell positions were preserved (see Materials and Methods). In R patients, the *Antigen presentation/Interferon* program strongly colocalized with B and T cells (Fig. 4E, top), reflecting an active immune response within these regions. This spatial proximity in a representative R patient (Fig. 4E, bottom) highlights regions of close proximity between *Antigen presentation/Interferon* tumor cells and lymphocytes, indicative of enhanced antigen processing and presentation that promotes T and B cell activation and effective antitumor immunity.

To systematically capture recurrent spatial arrangements, we developed an unsupervised computational pipeline to define *ecotypes*, i.e., recurrent spatial composition patterns of cellular states across tissue slides (see Materials and Methods). We identified five ecotypes named according to their cell-type composition as *Antigen-presentation/Inflamed*, *Hypoxia*, *Immune-stroma interface*, *Keratinocyte epithelium*, and *Tumor core* (fig. S6C). As shown in a representative specimen, Antigen-presentation/Inflamed ecotype dominated and intermixed with Immune-stroma interface regions, reflecting an activated immune microenvironment typical of R patients (fig. S6D). In contrast, NR tumors were dominated by Tumor core and Hypoxia ecotypes, coupled with stromal enrichment and limited immune infiltration (fig. S6E). Together, these observations suggest that identical cellular components can assemble into divergent ecosystem architectures, distinguishing responsive, inflamed tumors from resistant, immune-excluded, and stromal-dominated ones.

### TE reactivation drives immune signaling and contributes to tumor-immune dynamics

**Fig. 5.**
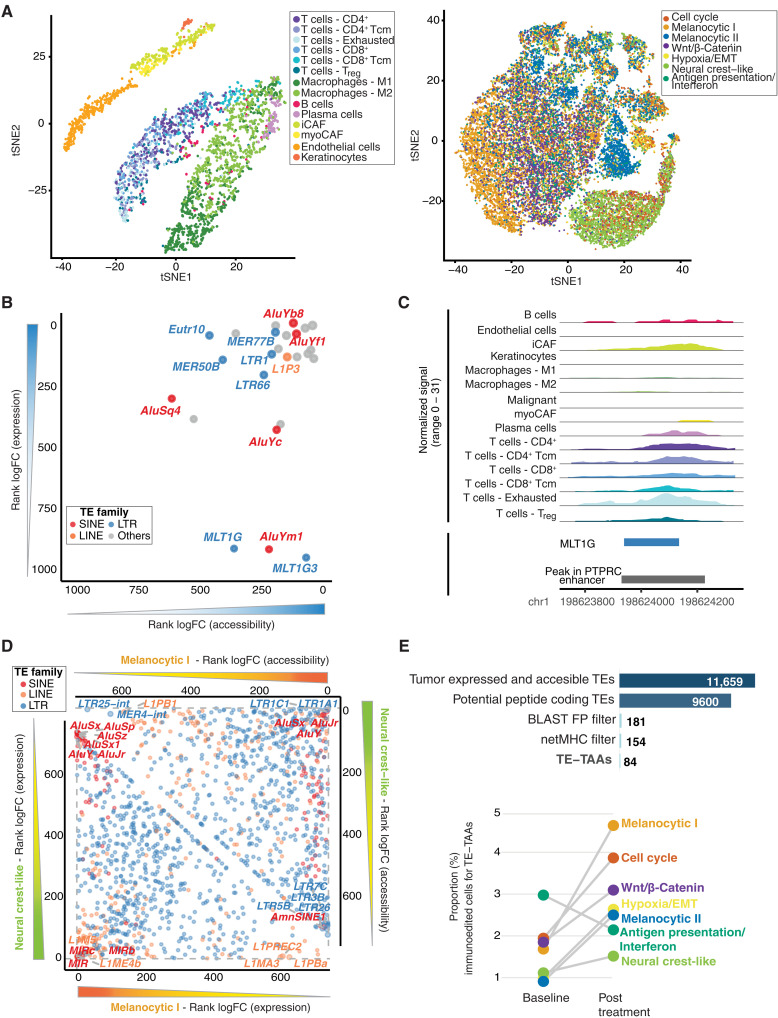
TE activity across tumor and TME states under epi-immunotherapy. (**A**) t-SNE of co-embedding of transposable elements (TEs) from paired snRNA-seq and snATAC-seq across tumor microenvironment (TME) cells (left) and tumor metaprograms (right). (**B**) Differential analysis of TE activity in TME cells pretherapy versus on-treatment (week 4), comparing chromatin accessibility (*x* axis) and expression (*y* axis), ranked by log_2_FC; points colored by TE family. (**C**) Accessibility of an MLT1G locus overlapping a *PTPRC* enhancer, showing normalized accessibility signal across all TME subtypes and malignant cells. (**D**) Comparative analysis of TE activity between *Melanocytic I* and *Neural crest–like* states, highlighting specific and shared TE patterns. Upper triangle: Chromatin accessibility fold-change ranks; lower triangle: expression fold-change ranks; features colored by TE family. (**E**) Top: Filtering strategy and number of TE-derived tumor-associated antigens (TE-TAAs). Bottom: Fraction of malignant cells classified as immunoedited, defined by concurrent TE expression and accessibility at loci generating patient-matched immunogenic TE-TAAs. Percentages of immunoedited cells were calculated per metaprogram and compared across treatment time points.

To evaluate the extent to which reactivated TEs contribute to shaping tumor and immune cell identities, as well as their interactions, we quantified TE activity across both malignant and nonmalignant compartments. Leveraging a multimodal integrative framework optimized for locus-specific quantification of TEs in single-cell sequencing (*60*), we profiled the expression and chromatin accessibility of major TE classes, including long interspersed nuclear elements (LINEs), short interspersed nuclear elements (SINEs), long terminal repeat elements (LTRs), and other repetitive elements. TE-derived signals proved highly informative for resolving the cellular architecture of the TME, with co-embedding t-SNE analysis separating stromal, T cell, and macrophage subpopulations (Fig. 5A, left). Moreover, TE activity reflected malignant cell heterogeneity across transcriptional MPs, revealing a continuum of tumor cell states rather than sharply segregated clusters (Fig. 5A, right).

We next assessed whether therapeutic intervention with demethylating agents modulated TE dynamics in the TME. Differential analysis of TME cells before therapy (week 0) versus on-treatment (week 4) revealed that DNMTi increased TE activity, evident at both the level of chromatin accessibility (*x* axis) and transcriptional expression (*y* axis) for specific TE elements (Fig. 5B and table S14). A subset of TEs showed simultaneous high accessibility and expression after treatment, including SINE Alu elements (e.g., *AluYb8* and *AluYf1*), LTR elements (e.g., *MER77B* and *LTR1*), and LINE-1 elements (e.g., *L1P3*), which emerged as top-ranking features in both RNA and accessibility analyses. Notably, Alu elements represent some of the most evolutionarily recent and transcriptionally active TEs in the human genome (mean ages of 1.89 and 5.36 Myr, respectively), suggesting that DNMTi treatment preferentially reactivates young, transcriptionally competent elements. In contrast, different TE families, such as LTR elements as *MLT1G* and *MLT1G3*, exhibited reactivation predominantly at the accessibility level, without corresponding transcriptional up-regulation. This pattern suggested a potential cis-regulatory role of such elements, whereby epigenetic reprogramming induced by DNMTi may prime these elements to act as regulatory enhancers, contributing to the transition of immune cells from a resting to an activated state in response to treatment. We indeed observed that a specific *MLT1G* locus (chr1: 198,623,845 to 198,624,639, GRCh38/hg38) overlapped an accessible chromatin region annotated as a *PTPRC* (CD45) enhancer and an active regulatory feature in CD45^+^ cells (Fig. 5C).

To dissect the contribution of TEs to malignant identities and transcriptional programs, we identified differentially expressed and accessible TEs across MPs (table S15). We first examined TE involvement along the melanoma differentiation axis, spanning from the highly differentiated *Melanocytic I* state to the stem-like, dedifferentiated *Neural crest*–*like* state (Fig. 5D).

While some TEs, including *LTR1C1*, *LTR1A1*, *AluSx*, *AluJr*, and *AluY*, were accessible across both states, the Alu elements, known for their role in viral mimicry and activation of innate immune sensing that may enhance responsiveness to epigenetic therapy (*61*), were selectively re-expressed in *Melanocytic I* tumor cells. We then extended this analysis to identify the most active TEs among the various metaprograms. Differential expression and accessibility analyses between conditions and rank area under the curve (AUC) values were computed independently from scRNA-seq and scATAC-seq data to quantify metaprogram-specific enrichment in expression and chromatin accessibility (fig. S7). Across metaprograms, distinct TE families displayed coordinated changes in chromatin accessibility and transcription. LTR elements, including multiple ERV and MER families, were preferentially associated with *Antigen presentation/Interferon* and *Hypoxia/EMT* programs, consistent with activation of viral mimicry-related pathways in stressed or dedifferentiated states. In contrast, LINE elements showed more selective and heterogeneous activation, with enrichment in proliferative and neural crest–like programs. SINE elements, particularly the Alu and MIR families, showed broad and consistent accessibility and expression across multiple metaprograms, suggesting a more pervasive role in transcriptionally active states. Moreover, while many TE families showed concordant accessibility and expression, discrepancies between the two modalities for specific elements point to additional post–chromatin regulatory mechanisms controlling TE transcription.

We then assessed whether reactivated TEs could also serve as a source of tumor-associated antigens (TE-TAAs), which are known to enhance immune responses under epigenetic interventions (*62*). Through a stringent computational workflow, we identified 84 high-confidence TE loci with potential to generate TAAs, corresponding to 154 unique TE-derived sequences with predicted antigenicity (Fig. 5E, top, and table S16). To determine whether the predicted TE-TAAs are effectively presented by HLA-I molecules, we generated a nonredundant set of 99 unique core peptide sequences (minimum 8-mer length) from the identified TE-derived sequence pool. We interrogated a collection of 31 mass spectrometry (MS)–based immunopeptidomics datasets from melanoma and other tumor types (*63*) and found that 37.4% of these peptides were confirmed by MS as HLA-I–presented ligands (table S17). These findings indicate that a substantial proportion of the predicted reactivated TE loci give rise to peptides that are naturally processed and presented by the HLA class I machinery, thereby supporting the bona fide antigenic potential of TE-derived TAAs. To quantify the fraction of malignant cells potentially subject to immune elimination through TE-TAAs, we defined as immunoedited those cells that simultaneously (i) displayed expression and chromatin accessibility at a TE locus potentially generating a TE-TAA, and (ii) matched an immunogenic TE-TAA identified in the corresponding patient. For each MP, we then calculated the percentage of immunoedited cells relative to the total malignant cells of that MP and compared their abundance across treatment time points (baseline at week 0 versus posttreatment at weeks 4 and 12). Notably, only tumor cells belonging to the *Antigen presentation/Interferon* state, previously characterized by strong antigen-presentation machinery and immunoproteasome gene expression, declined following treatment, consistent with a potential immune-mediated clearance driven by TE-derived antigens (Fig. 5E, bottom).

Altogether, these results highlight a dual role for TEs in melanoma under epi-immunotherapy: acting in cis as potential regulatory elements shaping immune activation, and in trans by generating immunogenic peptides that contribute to tumor-immune interactions, modulating both cell identity and tumor plasticity. Moreover, these results support the notion that one of the main mechanistic effects of the epigenetic therapy is to promote the transcription of TE-derived antigens and enhance antitumor immune response, as previously observed in glioma (*64*) and lung (*65*).

### NFATC2 drives resistance to ICI and epi-immunotherapy, and its targeting shifts neural crest–like melanoma toward a differentiated phenotype

Melanoma cells can shift from proliferative/differentiated states to invasive/dedifferentiated states, the latter being linked to poorer response to ICI therapy and reduced survival (*39*). To assess the clinical relevance of the transcriptional MPs identified in our single-cell analyses, we analyzed a large collection of eight previously published cohorts of patients with melanoma (table S18). These patients were treated with different ICI treatments, and their tumors were profiled by bulk transcriptomics before and/or after therapy. To minimize confounding effects from nonmalignant cells, we retained only high-purity tumor samples (purity ≥ 0.3, *n* = 402, R = 152, NR = 250).

**Fig. 6.**
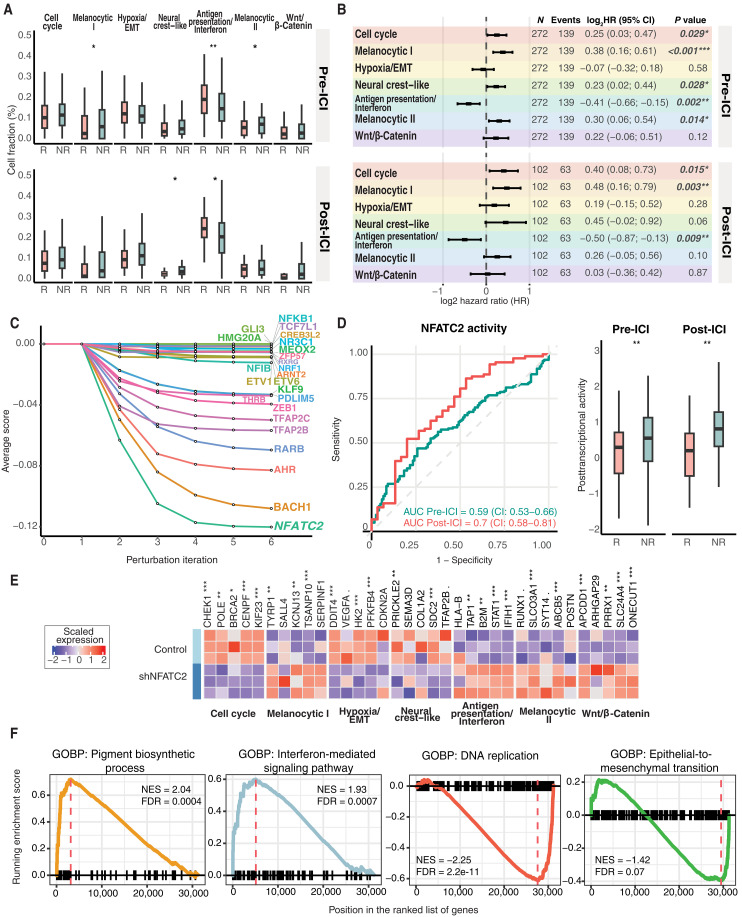
Prognostic value of metaprograms and regulatory role of NFATC2. (**A**) Boxplot showing MP deconvolution in bulk RNA-seq cohorts stratified by pre- and post-ICI treatment (pre-ICI: responders, Rs = 30, nonresponders, NRs = 88; post-ICI: Rs = 122, NRs = 162). Statistical significance was assessed using a two-sided Wilcoxon rank-sum test; *P ≤ 0.05, **P ≤ 0.01. (**B**) Forest plot of univariate Cox regression models for overall survival (OS) in pre-ICI (top) and post-ICI (bottom) bulk RNA-seq cohorts. Each MP score was modeled as a continuous variable (*z*-score standardized, per 1 SD increment). Hazard ratios (HRs) with 95% confidence intervals (CIs) are shown on a log_2_ scale; *P ≤ 0.05, **P ≤ 0.01, ***P ≤ 0.001. (**C**) Predicted changes in the *Neural crest–like* transcriptional state following simulated knockout of MP-associated TFs. For each TF, the mean difference in *Neural crest–like* signature gene expression across five independent iterations is shown. (**D**) Receiver operating characteristic (ROC) curves of response prediction in pre- and post-ICI based on NFATC2 posttranscriptional activity (left), and boxplots of activity scores stratified by responder status (R versus NR) before and after ICI (right). Areas under the ROC curve (AUCs) are shown with 95% CIs. Statistical significance was assessed using a two-sided Wilcoxon rank-sum test; **P ≤ 0.01. (**E**) Heatmap of scaled expression of selected genes in control and shRNA *NFATC2* conditions. Rows represent conditions and columns represent marker genes grouped by MP. Statistical significance of differential expression was assessed with limma; .*P* ≤ 0.1, *P ≤ 0.05, **P ≤ 0.01, ***P ≤ 0.001. (**F**) Enrichment plots of representative Gene Ontology Biological Processes terms after *NFATC2* knockdown.

Using a deconvolution approach based on MP-specific gene signatures (*66*), we estimated the proportion of malignant cells corresponding to each MP in the bulk tumor samples and evaluated differences according to the response group (Fig. 6A). Consistent with our findings and prior observations (*14*), tumors from Rs were significantly enriched for the *Antigen presentation/Interferon* state both before and after ICI treatment (Wilcoxon test, *P* value = 0.003 and 0.031, respectively), highlighting its predictive value for therapy response. Conversely, we found that the *Melanocytic I/II* states were more abundant in NRs before treatment (Wilcoxon test, *P* value = 0.017 and 0.025, respectively). Notably, the dedifferentiated *Neural crest–like* cells became specifically enriched in NRs after treatment (Wilcoxon test, *P* value = 0.015). Intriguingly, the relative abundance of these two distinct cell populations at single-cell resolution, the *Antigen presentation/Interferon* and *Neural crest–like* states, demonstrated a diagnostic performance in predicting therapy response (fig. S8A). A higher fraction of *Neural crest*–*like* cells was significantly associated with poorer overall survival (OS) both before and after ICI treatment (fig. S8B). Consistently, the scores of the *Melanocytic I/II* were significantly associated with a poorer OS before ICI [log_2_ hazard ratio (HR) = 0.38, *P* value <0.001, log_2_HR = 0.39, *P* value = 0.014] together with *Neural crest–like* (log_2_HR = 0.23, *P* value = 0.028) in a univariate Cox regression model, whereas *Antigen presentation/Interferon* was associated with a better OS (log_2_HR = −0.41, *P* value = 0.002, Fig. 6B); in addition, the *Cell cycle* MP, which was not associated with response, was also associated with poorer survival (log_2_HR = 0.25, *P* value = 0.029). After therapy, the *Antigen presentation/Interferon* showed the strongest positive association with survival (log_2_HR = −0.5, *P* value = 0.009). We also performed multivariate Cox proportional regression adjusting for age, gender, disease stage, and *BRAF*/*NRAS* mutational status where available (fig. S8C and table S19). In the pre-ICI setting, the *Antigen presentation/Interferon* metaprogram was independently associated with improved OS after adjustment for age and gender (log_2_HR = −0.68, *P* value <0.001), an association that persisted after inclusion of disease stage (log_2_HR = −0.62, *P* value = 0.025). The *Neural crest*–*like* program was associated with worse outcomes in the age- and gender-adjusted model (log_2_HR = 0.41, *P* value = 0.003). In the post-ICI setting, the *Antigen presentation/Interferon* program showed a strong protective association (log_2_HR = −1.19, *P* value = 0.002), also after stage adjustment (log_2_HR = −1.51, *P* value = 0.005). The *Melanocytic I* metaprogram was consistently associated with worse OS in the post-ICI setting across all model specifications (log_2_HR = 1.20, *P* value <0.001; with stage: log_2_HR = 1.25, *P* value = 0.005), suggesting that a differentiated melanocytic transcriptional state may confer reduced benefit from immune checkpoint blockade. Continuous and discrete (median-stratified high versus low) univariate and multivariate models are reported in table S19.

Our previous finding showed that melanoma *Neural crest–like* cells exhibit a lower level of plasticity than other melanoma cells; therefore, we explored potential molecular drivers of these therapy-resistant transitions. We performed a computational perturbation analysis using SCENIC+ (*67*) to determine how perturbing the expression of key master regulator TFs would affect this cell state (Fig. 6C). Among the candidates, simulated silencing of *NFATC2* produced the most pronounced effect, nominating it as a central regulator of the resistant *Neural crest–like* state.

To characterize *NFATC2* function, we inferred its regulon from the independent single-cell dataset of patients with melanoma used before (*14*). This approach was crucial for minimizing potential bias from our own epigenetic therapy dataset, allowing us to define a high-confidence *NFATC2* regulon, consisting of 56 genes (fig. S9A). As expected for a program sustaining a *Neural crest–like* phenotype, this regulon contained a number of genes associated with embryonic development, invasion, migration, and neurogenesis. Among these genes, we found key drivers of developmental mechanics, such as the AP-2 family members *TFAP2C* and *TFAP2B*, which are critical for the development of ectodermal tissues and the neural crest (*68*). We also identified genes that are key drivers of tumor cell invasion and migration, including *CSPG4* and *CAV1*, genes previously associated with tumor cell migration, poor prognosis, and multidrug resistance in cancer (*69*, *70*). As anticipated, the regulon also contained genes with established roles in neurogenesis and neuronal function, such as *MAP2* and *SEM3D*, markers of neuronal differentiation, cell migration, and axon growth (*71*, *72*). The transcriptional activity of the *NFATC2* regulon predicted secondary resistance to ICI therapy (AUC = 0.7, Fig. 6D), with significantly higher activity in NR samples both before and after therapy (Wilcoxon test, *P* value = 0.007 and 0.001, respectively).

To connect our in silico findings with experimental evidence, we analyzed stable *NFATC2* knockdown data in a melanoma cell line derived from a surgical specimen (*73*–*75*). The cell line, Me71, was identified through a focused screening for *NFATC2* expression in panels of melanoma cell lines isolated from surgical specimens of patients with metastatic melanoma (*74*). It constitutively expresses several molecules associated with a melanoma dedifferentiated state (*74*). We confirmed that Me71 expresses higher levels of Neural crest–like and Neural crest–like transitory programs and shows a lower activation of melanocytic differentiation programs (fig. S9B). By overlapping the top 50 differentially expressed genes (DEGs) of each MP (table S5) with DEGs from *NFATC2*-silenced cells (table S20), we found robust inactivation of both *Cell cycle* and *Neural crest–like* programs (fig. S9, B and C). Key down-regulated genes included *CHEK1*, *POLE*, and *CENPF* (*Cell cycle*) and *PRICKLE2*, *SDC2*, and *TFAP2B* (*Neural crest–like*) (Fig. 6E). Gene set enrichment confirmed suppression of DNA replication and EMT transitions, accompanied by an increase of interferon-mediated signaling and pathways associated with melanocyte differentiation, such as pigment biosynthetic process (Fig. 6F and table S21). Conversely, we observed a transcriptional shift toward a more differentiated *Melanocytic II* state (Fisher’s method, *P* value = 4.4 × 10^−4^, fig. S9C), with reactivation of pigmentation pathways, and simultaneously activated interferon-mediated signaling, including up-regulation of specific genes of the *Antigen presentation/Interferon* MP such as *TAP1*, *B2M*, *STAT1*, and *IFIH1*.

Together, these findings establish *NFATC2* as a key regulator of the *Neural crest*–*like* melanoma state, coupling proliferative and migratory programs with therapy resistance. Silencing of *NFATC2* not only reverted this resistant state but also promoted differentiation and immunogenic reprogramming, highlighting *NFATC2* as a potential therapeutic vulnerability in aggressive melanoma. The *NFATC2*-driven program may also contribute to mechanisms of resistance against epi-immunotherapy, positioning its inhibition as a strategy to enhance responsiveness to such treatments.

## DISCUSSION

Our multiomic and spatial dissection of longitudinal tumor biopsies provides a mechanistic framework for how epi-immunotherapy reshapes the melanoma ecosystem. We identify tumor cellular states and regulatory programs that underlie therapeutic sensitivity and resistance, revealing actionable vulnerabilities. Prior bulk and single-cell studies established the existence of discrete melanoma cellular states, such as MITF^high^ melanocytic, AXL^high^ invasive, stress-related, antigen-presenting, and neural crest–like, linked to therapy response and resistance in ICI therapies (*1*, *3*, *12*, *13*). We confirm these states but, crucially, show how they evolve dynamically under combined DNMT1 inhibition and CTLA-4 blockade. In particular, we uncover substantial heterogeneity within the melanocytic compartment, resolving three distinct transcriptional programs. One of these programs is uniquely characterized by activation of the Wnt/β-Catenin signaling pathway, evidenced by strong expression of *LEF1*. This represents a previously unappreciated melanocytic substate with potential relevance for immune evasion and therapeutic resistance (*76*). The persistence of a *Neural crest–like* program in nonresponders and enrichment of an *Antigen presentation/Interferon* program in responders parallels recent observations of phenotype switching as a mechanism of immune evasion (*14*), while adding mechanistic depth from paired transcriptome-chromatin profiling.

Several studies have shown that immunotherapy can induce melanoma dedifferentiation, often via IFN-γ/TNF-driven inflammation, leading to loss of melanocytic markers and adaptive resistance (*41*–*43*). While we did not observe a simple association between dedifferentiation and resistance, comparative analyses revealed that the *Antigen presentation/Interferon* metaprogram, the state most strongly associated with response, displayed one of the highest stemness scores, comparable to the *Neural crest*–*like* program. Thus, in our dataset, response-associated states overlap with stemness and dedifferentiation features rather than representing a strictly differentiated melanocytic phenotype. Consistent with our recent multiomics study (*6*), in which epigenetically defined dedifferentiated melanomas were enriched for immune features and associated with improved relapse-free survival under adjuvant checkpoint blockade, these findings indicate that dedifferentiation is not uniformly a resistance mechanism. Instead, its clinical impact appears to depend on the accompanying epigenetic and transcriptional context. A possible interpretation is that melanoma plasticity generates biologically distinct dedifferentiated states, some linked to immune escape, others compatible with, or even supportive of, immunotherapy responsiveness.

Our study highlights the critical role of spatial organization in shaping melanoma cellular states and their functional stability under epi-immunotherapy. By integrating TSPS analysis with trajectory-based transition modeling, we demonstrate that clustered tumor cells exhibit both greater transcriptional homogeneity and a higher probability of maintaining their state and spatial configuration compared to dispersed counterparts. These findings indicate that spatial clustering reinforces cell state identity, whereas dispersion promotes transcriptional heterogeneity and state instability. This principle has been recently described in other cancer contexts (*15*, *77*), while studies of circulating tumor cell clusters have demonstrated that homotypic adhesion supports survival and functional stability during metastasis (*78*, *79*). Collectively, these observations support a concept in which differential homotypic adhesion acts as an organizing principle in cancer, preserving cellular identity in clustered niches and enabling plasticity in dispersed cells. The unusual bimodal TSPS distribution of the *Wnt/*β*-Catenin* program provides a further example of the relationship between pathway biology and spatial architecture. β-Catenin, at adherens junctions, stabilizes cadherin-mediated homotypic adhesion, fostering cohesive architecture, whereas in the nucleus, it functions as a transcriptional co-activator, driving programs linked to plasticity, invasion, and immune evasion (*80*). The coexistence of clustered and dispersed *Wnt/*β*-Catenin* subpopulations may therefore reflect these two roles of β-Catenin. Clustered cells likely exploit adhesion-mediated stabilization to maintain coherence, while dispersed cells may engage β-Catenin’s transcriptional activity, resulting in greater heterogeneity and state transitions. We also observed that *Antigen presentation/Interferon* and *Hypoxia/EMT* programs displayed the highest homotypic transition probabilities. These states may represent functionally reinforced niches, where spatial clustering enhances stability and ensures persistence under therapeutic pressure. Conversely, dispersed cells across states showed lower self-transition probabilities, consistent with their role as reservoirs of plasticity and adaptation. Together, our findings reveal a model in which spatial clustering and dispersion constitute parallel organizational strategies in melanoma: Clustered niches, stabilized by homotypic adhesion, preserve state identity and may contribute to treatment resistance, while dispersed cells provide a source of heterogeneity and adaptive potential.

Our results support and extend prior work showing that epigenetic drugs can enhance responsiveness to ICIs by inducing viral mimicry and innate immune signaling (*61*). We demonstrate that TE reactivation has a dual role: acting as CREs that prime immune activation, and in trans, generating TE-derived antigens cleared by T cells. While similar TE-mediated immunogenicity has been reported in other cancers (*64*, *65*), our study provides single-cell resolution evidence that TE-driven antigenicity actively shapes melanoma cell fate under epi-immunotherapy. We uncover an inverse association between TE reactivation and the *Antigen presentation/Interferon state*, supporting the hypothesis that tumor immuno-editing in the context of epigenetic therapy is highly dependent on such cellular state. This highlights TE reactivation not merely as a global effect of treatment, but as a state-specific mechanism that contributes to immune pressure and fate decisions in melanoma.

Prior studies have implicated *NFATC2* in EMT-like transitions and resistance in MITF^low^ melanoma (*74*, *75*). We identify *NFATC2* as a master regulator of a specific therapy-resistant cellular state linking invasive behavior and immune evasion. Our findings directly associate *NFATC2* in driving the Neural crest–like phenotype itself, providing a mechanistic link between cell state and therapeutic failure. These results are consistent with prior studies showing that, in MITF^low^ melanomas, *NFATC2* regulates the EMT-like transcriptional program, invasive potential, and tumor growth in vitro and in vivo, in part via oncogenic networks involving *c-MYC*, *FOXM1*, and *EZH2* (*73*–*75*). This positions *NFATC2* inhibition as a rational therapeutic strategy to overcome both primary and acquired resistance to epi-immunotherapy.

One of the main limitations of our study is the small number of patients and limited sampling time points/technical challenges, such as obtaining sufficient high-quality tissue inherent to proof-of-concept trials; therefore, larger studies will be required to generalize some of our observations (*81*). Our study relied on computational and orthogonal validation strategies to support the biological relevance of the inferred regulatory networks and predicted TAAs. While these multilayered approaches provide converging lines of evidence, we recognize that further validation using prospectively collected samples and direct experimental assays will be essential to translate these findings toward clinical applications. Moreover, functional validation of the predicted TE-derived TAAs would confirm their immunogenic potential and clinical relevance. Nevertheless, together, our results establish a framework in which epi-immunotherapy reshapes melanoma ecosystems by promoting immune-engaged states while exposing vulnerabilities of resistant niches. Through the integration of single-cell multiomics and spatial analyses, we provide mechanistic insights into tumor plasticity, TE-mediated immune dynamics, and regulatory drivers of resistance.

## MATERIALS AND METHODS

### Ethics statement

The phase 1b NIBIT-M4 study (NCT02608437) was conducted in accordance with the ethical principles of the Declaration of Helsinki and the International Conference on Harmonization of Good Clinical Practice. This study complies with all ethical regulations as per the Ethical Committee “Comitato Etico regione Toscana - Area Vasta Sud-Est” evaluation and approval report CE 61/2015. All participants provided informed, written consent.

### Experimental method details

#### Sample collection

Snap-frozen tumor tissues (*n* = 13) were collected from five patients with advanced cutaneous melanoma enrolled in the phase 1b NIBIT-M4 clinical trial (NCT02608437), as previously described (*9*). Briefly, patients received escalating doses of the DNA hypomethylating agent guadecitabine (30 to 60 mg m^−2^ day^−1^, subcutaneously on days 1 to 5) in combination with the standard anti–CTLA-4 ipilimumab (3 mg/kg, intravenously on day 1) across four treatment cycles. Tumor biopsies for correlative analyses were collected longitudinally at baseline (week 0), during treatment (week 4), and at a later on-treatment time point (week 12). Patients were classified as responder (R; complete response, partial response, or stable disease) or nonresponder (NR; progressive disease) patients, based on clinical response.

#### *10x Genomics single-nucleus multiome* (*RNA + ATAC*) *sequencing*

Nuclei were isolated from 13 snap-frozen tissues using the Nuclei isolation kit (10x Genomics, CG000505 Rev. A) following the manufacturer’s instructions. Nuclei counts and viability were assessed using Nexcelom Cellometer K2 (Nexcelom Bioscience). A range of 3000 to 10,000 nuclei per sample was used for single-cell multiome libraries generation according to the Chromium Next GEM Single cell Multiome ATAC + Gene Expression (10x Genomics, CG000338 Rev. F). Briefly, transposed nuclei were combined with barcoded Gel Beads, a Master Mix, and Partitioning Oil on a Chromium Next GEM Chip J for gel bead-in-emulsions (GEM) generation and barcoding. GEX and ATAC libraries were processed separately, and their quality was examined on a 5200 Fragment Analyzer (Agilent Technologies). Sequencing was performed on Novaseq X Plus (Illumina).

#### Visium HD spatial gene expression profiling

Formalin-fixed paraffin-embedded (FFPE) tissues were reviewed by a board-certified pathologist and placed on the Visium slide following the Visium CytAssist Spatial Gene Expression for FFPE – Tissue Preparation Guide (10x Genomics, CG000518, Rev. C). Spatial gene expression libraries were generated following the Visium HD Spatial Gene Expression Reagent Kits (10x Genomics, CG000685, Rev. A). Sequencing was performed on Novaseq X Plus (Illumina).

### Data processing and statistical analyses

#### Single-nucleus multiome data processing

The 10x Genomics Cell Ranger ARC (v2.0.2) pipeline was used to process the multiome data. The GRCh38 human reference was used for the read mapping (refdata-cellranger-arc-GRCh38-2020-A-2.0.0). The raw RNA count matrix and ATAC fragment data were further processed using R packages Seurat (v5.1.0) and Signac (v1.14.0), respectively.

##### RNA-seq modality

Low-quality nuclei were excluded from the RNA-seq dataset based on standard QC metrics. Specifically, for each sample, nuclei exhibiting either low or excessively high gene complexity (<500 or >7500 detected genes), elevated mitochondrial gene content (>5%, indicative of dying or stressed cells), or abnormally high total transcript counts (nCount >50,000) were filtered out. Genes detected in at least three nuclei were retained. After QC filtering, a total of 58,670 high-quality nuclei were retained for downstream analyses [median 3675 nuclei per sample, with 5722 unique molecular identifiers (UMIs) and 2774 genes detected per nucleus].

The merged gene expression matrix was subsequently normalized and log transformed. Dimensionality reduction was performed via principal components analysis (PCA) using the top 3000 highly variable genes. The first 22 principal components (PCs) were then used to construct t-SNE for visualization, and unsupervised clustering was carried out using a resolution of 0.2.

##### ATAC-seq modality

Raw ATAC-seq fragment data were processed by merging the common peaks across samples using the *reduce* function from the GenomicRanges package (v1.54.1). Peaks shorter than 20 or longer than 10,000 base pairs were excluded as outliers.

Nuclei with low-quality chromatin accessibility profiles were excluded based on standard QC criteria. Specifically, nuclei with fewer than 2500 or more than 20,000 total fragments were removed. Additional exclusion criteria included a low fraction of fragments in peaks (percent reads in peaks <20%), a high proportion of reads mapping to ENCODE blacklisted regions (*82*) (blacklist ratio > 0.05), elevated nucleosome signal (>1.5), and low transcription start site (TSS) enrichment (<2). Following QC filtering, a total of 55,850 high-quality nuclei were retained for downstream analysis (median of 3944 nuclei per sample, with 16,472 fragments per cell and a TSS enrichment of 3.83).

The resulting peak count matrix was normalized using term frequency–inverse document frequency transformation. Dimensionality reduction was performed via singular value decomposition (SVD) on the top 95% most commonly accessible peaks (*FindTopFeatures with min.cutoff set to “q5”*). The latent semantic indexing (LSI, 2:50) components from the SVD reduction were used for secondary dimensionality reduction via t-SNE. The first LSI component, which was strongly correlated with sequencing depth, was excluded from downstream analyses. Unsupervised clustering was performed using the smart local moving (SLM) algorithm implemented in the Seurat package (*FindClusters* with *algorithm* = 3, at a resolution of 0.8). Peak calling was conducted using the MACS2 package (v2.2.6) via the *CallPeaks* function. Gene activity scores were computed using the *GeneActivity* function, which aggregates chromatin accessibility across gene bodies and promoter regions.

##### Integrating modalities

To integrate information from both RNA and ATAC modalities for multiomic clustering, nuclei passing QC filters in both datasets (*n* = 38,029) were retained. The *FindMultiModalNeighbors* function in Seurat was used to construct a Weighted Nearest Neighbor (WNN) graph by jointly considering the RNA PCs (1:14) and ATAC latent semantic indexing components (LSI 2:50). This WNN graph was used as input for dimensionality reduction using t-SNE. Unsupervised clustering was subsequently performed on the Weighted Shared Nearest Neighbor graph using the SLM algorithm with a resolution parameter set to 0.1.

#### Malignant and nonmalignant cell discrimination and TME cell-type annotation

Classification of cells into malignant and nonmalignant cells was performed individually on RNA modality using the SCEVAN package (v.1.0.3) (*16*), which integrates single-cell expression data with inferred CNAs to identify malignant clones. Following malignancy assignment, nonmalignant cells were clustered using the first 12 PCs and a resolution of 0.7. Subsequently, cell types were annotated using well-established immune and stromal cell-type marker signatures (*83*), combined with the inspection of canonical marker genes at both the gene expression and chromatin accessibility levels. To systematically evaluate immune cell-type enrichment across clusters, we applied the Normalized Enrichment Score derived from the Mann-Whitney-Wilcoxon Gene Set Test (MWW-GST), as previously described in (*84*), on gene ranks obtained by differential expression analysis between each Seurat cluster and all others. On the basis of this integrative analysis, the TME was resolved into distinct cellular subtypes, including components of the immune compartment: T cells (CD8^+^, CD4^+^, exhausted, and T_reg_ cells), B cells, plasma cells, and macrophages (M1 and M2 subtypes), as well as the stromal compartment, comprising CAFs (iCAFs and myoCAFs), endothelial cells, and keratinocytes.

#### Malignant cell MP identification

Malignant cell MPs were identified following the approach described by Gavish *et al.* (*18*). Briefly, non-NMF was independently applied to the relative gene expression profiles of malignant cells from samples containing at least 300 malignant cells. Before NMF, all negative expression values were set to zero. To avoid predefining the number of transcriptional programs (rank *k*), NMF was run across multiple values (*k* = 4, 5, …, 9), yielding a total of 468 NMF programs. Each NMF program was defined by the top 50 genes with the highest NMF coefficients. To ensure biological relevance and consistency, only robust and nonredundant NMF programs were retained. Robustness was defined by high intrasample consistency (minimum 70% gene overlap across different k values), low redundancy within samples (maximum 20% overlap), and limited overlap across patients (maximum 20%). This filtering resulted in 75 robust NMF programs. These programs were subsequently grouped based on pairwise Jaccard similarity using an iterative hierarchical clustering approach that merged highly overlapping programs, as previously described (*18*). Each resulting MP was defined by the 50 most recurrent genes across the NMF programs within each cluster.

#### Classification of malignant cells into MP states and functional characterization

Each malignant cell was scored for all seven MP states using a gene set scoring approach. Specifically, the *AddModuleScore* function from the Seurat package was used to compute a signature score for each MP based on its corresponding 50-gene set. Cells were retained for further classification only if their highest MP signature score exceeded 0.3.

To quantify the confidence in assigning a cell to a specific MP state, a simplicity score was computed as follows

$$\text{Simplicity score}=\frac{\text{Highest}\;\text{MP}\;\text{score}-\text{Second highest}\;\text{MP}\text{score}}{\text{Highest}\;\text{MP}\;\text{score}}$$

Cells with a simplicity score ≥ 0.1 were confidently assigned to the MP corresponding to their highest score. We observed that the majority of unclassified cells exhibited competing scores for *Melanocytic I* and *Melanocytic II*, or the *Wnt/*β*-Catenin* program. Because these cells do not exhibit a distinct, independent transcriptional signature and instead fall between established melanocytic programs, we interpret them as representing transitional or intermediate states along the melanocytic differentiation continuum.

To assess the biological relevance of each MP, functional enrichment analyses were applied. Overrepresentation analysis (hypergeometric test) was conducted on the 50-gene MP signatures. In parallel, Gene Set Enrichment Analysis was performed using the clusterProfiler package (v4.10.1) on ranked gene lists derived from differential expression between each MP and all other malignant cells, as computed with the memento framework (v0.1.1). Curated gene sets were obtained from Gene Ontology (GO), Reactome, Kyoto Encyclopedia of Genes and Genomes (KEGG), Hallmark, and WikiPathways databases. In addition, DEGs for each MP were defined using the thresholds of fold change (de_coef ≥ |ln (*2*)|) and adjusted *P* value <0.05. Cell-cell communication analysis was performed to evaluate interactions between malignant cells (stratified by MP assignment) and TME cell populations using the CellChat package (v2.1.2).

#### Cross-modal concordance analysis

Concordance between transcriptional and chromatin accessibility states was assessed by performing cross-modal label transfer with Seurat (v5.1.0) (*85*). The snRNA-seq dataset was used as the reference, and gene activity scores from the snATAC-seq dataset were used as the query. Cross-modal anchors were identified using canonical correlation analysis (CCA) with *FindTransferAnchors*, and labels were transferred with *TransferData* (LSI 2:50). Predicted annotations were assigned to individual snATAC-seq nuclei, compared with their reference states, and prediction scores were averaged across cell types to evaluate correspondence between transcriptional identities and accessibility-based predictions.

#### Comparison with melanoma gene signatures and functional states

Transcriptional metaprograms were compared with published melanoma gene signatures (*1*–*3*, *12*–*14*, *36*–*38*) using module scoring and concordance assessment by Pearson correlation. Metaprogram-specific pseudobulk profiles were further used to evaluate stemness and dedifferentiation functional states. Specifically, stemness levels were quantified using the top 100 genes from the SC_PCBC_stemSig stemness signature from TCGABiolinks (v2.30.4) described by Malta *et al.* (*40*), while dedifferentiation was assessed using both an IFN-γ–induced dedifferentiation signature (*41*) and a minimal marker–based index defined as *NGFR* expression relative to the mean expression of *PMEL* and *MLANA* (*42*). Scores for all gene signatures and functional states were computed using Seurat’s *AddModuleScore* function, and differences between metaprograms were tested using a two-sided Wilcoxon rank-sum test.

#### TF motif enrichment analysis

TF motif enrichment was analyzed on DA peaks for each metaprogram relative to all others (average log_2_FC ≥ 0.58 and adjusted *P* value < 0.05). To mitigate sequence composition bias, accessible peaks were matched to background regions with comparable GC content using *MatchRegionStats* function from Signac. Enrichment was tested with *FindMotifs* hypergeometric framework from Signac, using TF position weight matrices from the HOCOMOCO v13 database. Motifs with fold enrichment ≥ 1.3 were retained. To avoid redundancy, TFs represented by multiple motifs were filtered such that only the most strongly enriched motif per TF was kept. The union of the top 30 TFs per metaprogram, along with *MITF*, was displayed as a heatmap, with values representing fold enrichment over background. Motif footprinting for selected TFs was performed with the *Footprint* function from Signac using the GRCh38/hg38 genome assembly.

#### *Cis-regulatory region* (*CRE*)*–TFs-gene linkage analysis*

Genes linked to enriched TF motifs (as described above) were mapped to PLS, pELS, and dELS based on ENCODE candidate cis-regulatory element (cCRE) annotations. For each regulatory category, motif-containing cCREs were intersected with their linked protein-coding genes, and only those also identified as up-regulated from DEG analysis between each MP versus the others were retained. These filtered gene sets were then subjected to overrepresentation analysis using the *enricher* function from clusterProfiler (*86*), using curated gene sets from databases including GO biological process categories, Reactome, KEGG, and Hallmark.

#### eGRN analysis

eGRNs were inferred from snRNA-seq and snATAC-seq multiome data using SCENIC+ (v1.0a2) (*64*). First, pycisTopic (v2.0a0) was used to identify and binarize cis-regulatory topics from the snATAC-seq dataset, and pycisTarget (v1.1) was used to identify enriched motifs. The SCENIC+ Snakemake pipeline was executed with default parameters to predict enhancer-driven regulons constituting the eGRN. Regulon specificity scores were calculated for each MP. High-quality eRegulons were defined by computing the correlation between TF expression and the activity scores (AUC) of their predicted target regions, retaining only TF-region pairs with correlation coefficients above |0.3|, which yielded 188 eRegulons with a median of 196 genes and 292 regions each. eRegulon activity was quantified using AUCell scores. TFs were subsequently ranked based on (i) TF expression in a given MP relative to all other MPs, (ii) eRegulon AUCell scores on gene expression in a given MP relative to all other MPs, and (iii) eRegulon AUCell scores on accessibility in a given MP relative to all other MPs. Only TFs ranked within the top 30 across all three criteria (*n* unique TFs = 104) were retained for downstream analyses. SCENIC+-derived regulons were then applied to external datasets, including an independent scRNA-seq melanoma cohort (*14*) and two additional samples from our cohort not used for eGRN inference, to assess TF specificity across metaprograms. Regulon activity was quantified using AUCell scores.

To assess the concordance between the enhancer regions inferred by SCENIC+ and known enhancer regions, ENCODE cCRE annotations were downloaded from the SCREEN Registry Version 3 (https://screen.encodeproject.org/), comprising enhancer-like elements across 49 tissues. The overlap was quantified both globally and across individual tissues and further stratified by enhancer class (pELS and dELS) using the subsetByOverlaps function of the GenomicRanges (v1.54.1).

#### ChIP-seq enrichment analysis

DA chromatin peaks associated with each of the seven transcriptional MPs were submitted as regions of interest to the Enrichment Analysis module of ChIP-Atlas (https://chip-atlas.org/) (*87*), a data-mining suite integrating more than 433,000 publicly available ChIP-seq experiments. Enrichment of TF binding was assessed by Fisher’s exact test with Benjamini-Hochberg multiple testing correction; only features with *Q* < 0.05 were retained for downstream analysis. Results across all seven MPs were jointly visualized as a bubble plot, in which the *y* axis displays the −log(*Q* value), bubble size is proportional to the number of supporting ChIP-seq experiments, and melanoma-derived samples are distinguished from other cell types.

#### Visium HD data processing

##### Nuclei segmentation and binning

We performed nuclei segmentation on the high-resolution H&E-stained images to delineate individual nuclei, enabling the assignment of spatial barcodes to specific cells. In detail, a pretrained deep learning model, StarDist (*88*), was used to create a nuclei mask; the shape and location of each nucleus in the mask were then used to sort barcodes into bins corresponding to each individual nucleus. Last, we aggregated gene expression counts from the 8 μm × 8 μm barcoded squares associated with each segmented nucleus by performing gene-wise summation of UMI counts, resulting in a gene-by-cell matrix approximating single-cell resolution. This procedure was independently repeated for each of the nine samples, resulting in an average of 113,366 cells per sample with 18,085 genes.

##### Data preprocessing

For each sample, low-quality cells and genes were removed. Specifically, we filtered out cells showing high mitochondrial gene content (>5%) or expressing fewer than five detected genes. In addition, genes expressed in fewer than five cells were also removed to reduce noise and focus on biologically relevant signals. A total of 736,028 cells passed the QC step, and their gene expression counts were normalized using total-count normalization to a target sum of 10,000 transcripts per cell, followed by log_2_ transformation. The data were then scaled to unit variance and zero mean for each gene.

##### Malignant and nonmalignant cell discrimination

Cells were clustered using Louvain’s algorithm with a resolution of 0.5. We performed differential expression analysis across clusters and used known tumor marker genes to classify cells as malignant or nonmalignant.

##### OT for annotation transfer between RNA-seq and Visium HD

OT is a mathematical framework that originates from the problem of transferring one distribution of mass to another in the most cost-efficient manner. In this work, we used the Fused Gromov-Wasserstein distance coupled with spatial constraints (*57*, *58*, *89*–*91*), to learn a probability matrix $\prod_{+}\in {\mathbb{R}}^{n_{1}\times n_{2}}$ whose positive entry $\prod_{\mathrm{ij}}$ denotes the probability of transporting cell i to cell $l_{j}$, between two data modalities composed of $n_{1}$ and $n_{2}$ cells, respectively. Given a pairwise alignment matrix $\prod =\left[\pi_{i,j}\right]$, mapping RNA-seq to Visium HD, we assigned to each nonmalignant Visium HD cell the cell-type label of the most probable match from the RNA-seq data. The same procedure was repeated to assign MP labels to malignant Visium HD cells.

##### Malignant and nonmalignant spatial interactions

To quantify spatial enrichment of malignant MPs and nonmalignant cells, we developed a tumor neighborhood analysis framework based on Euclidean spatial distances between cell coordinates. For each nonmalignant cell i, we first identified malignant neighbors within a fixed radius r. Let $N_{1}$, denote the set of malignant neighbors of cell i, and let T be the set of malignant subtypes. For each subtype t∈T, we computed the number of neighbors of subtype t as $c_{i}^{\left(t\right)}=\sum_{j\in N_{1}}1\left\{l_{j}=t\right\}$, where $l_{j}$ is the subtype label of neighbor $l_{j}$, and 1{⋅} is the indicator function. Aggregating these counts across nonmalignant cells resulted in a tumor neighbor count matrix stratified by subtype. To assess statistical significance, we conducted permutation testing by shuffling the malignant subtype labels uniformly at random N=100 times while preserving cell positions. For each permutation n, we recomputed neighbor counts $c_{i}^{\left(t,n\right)}$ and calculated the mean $\mu_{\text{perm}}^{\left(C,t\right)}$ and SD $\sigma_{\text{perm}}^{\left(C,t\right)}$ of neighbor counts for each nonmalignant cell type C. The observed mean for cell type C and tumor subtype t, denoted $\mu_{\text{obs}}^{\left(C,t\right)}$, was then compared to the null distribution using a *z*-score

$$z^{\left(C,t\right)}=\frac{\mu_{\text{obs}}^{\left(C,t\right)}-\mu_{\text{perm}}^{\left(C,t\right)}}{\sigma_{\text{perm}}^{\left(C,t\right)}}$$

##### Peripheral localization index

To quantify the spatial localization of malignant subtypes within the tissue architecture, we computed a PLI, measuring the tendency of a cell cluster to localize near the tissue boundary. In detail, let ${\left\{x_{i}\in {\mathbb{R}}^{2}\right\}}_{i=1}^{N}$ be the spatial coordinates of all cells, and let C⊆{1,…,N} denote the indices of cells in a target cluster. The boundary of the tissue is defined by the convex hull C⊆{1,…,N} of all cell coordinates. Let H be the coordinates of the convex hull vertices. We first computed the average distance to the boundary as

$$D_{\text{obs}}=\frac{1}{|C|}\sum_{i\in C}{\text{min}}_{j=1,\ldots ,M}{\left\|x_{i}-b_{j}\right\|}_{2}$$

To construct a null distribution, we generated K=100 random samples $R^{\left(1\right)},\ldots ,R^{\left(K\right)}$, each containing ∣C∣ cells drawn uniformly without replacement from the full population. For each sample, we then computed

$$D^{\left(k\right)}=\frac{1}{|C|}\sum_{i\in R^{\left(k\right)}}{\text{min}}_{j=1,\ldots ,M}{\left\|x_{i}-b_{j}\right\|}_{2},\;\text{for}\;k=1,\ldots ,K$$

The mean and SD of the null distribution are then

$$\mu_{\text{rand}}=\frac{1}{K}\sum_{k=1}^{K}D^{\left(k\right)},\sigma_{\text{rand}}=\sqrt{\frac{1}{K-1}\sum_{k=1}^{K}{\left(D^{\left(k\right)}-\mu_{\text{rand}}\right)}^{2}}$$

The PLI for a target cluster is lastly defined as

$$\text{PLI}=\frac{D_{\text{obs}}-\mu_{\text{rand}}}{\sigma_{\text{rand}}}$$

A negative PLI indicates that the cluster is significantly closer to the tissue boundary than expected by chance (i.e., peripheral enrichment), while a positive PLI suggests central localization.

##### Tumor state proximity score

To evaluate spatial clustering of phenotypically distinct tumor states, we computed a TSPS. For each tumor cell i, we defined its neighborhood $N_{1}$ as the set of cells located within a radius r in Euclidean space. Let $S_{i}\in S$ denote the tumor state label of cell i, and $S_{j}$ be the label of neighbor $j\in N_{1}$. The per-cell TSPS is defined as

$${\text{TSPS}}_{i}=\frac{1}{|N_{1}|}\sum_{j\in N_{1}}1\left\{S_{j}=S_{i}\right\}$$

where 1{⋅} is the indicator function. For each tumor state S∈S, we computed the average TSPS across all cells labeled with that state: $T\bar{\text{SP}}S_{S}=\frac{1}{|C_{s}|}\sum_{i\in C_{s}}{\text{TSPS}}_{i}$, where $C_{s}=\left\{i:S_{i}=S\right\}$ is the set of all cells assigned to state S.

##### Tumor region identification

For each sample, we delineated the tumor region directly from malignant cell coordinates. First, we estimated a continuous spatial intensity field using Gaussian KDE. The resulting density map was binarized with an adaptive threshold proportional to the mean density (τ·μ; τ = 0.5 by default). To regularize the mask, we removed small objects and holes and applied a binary closing followed by dilation. We then extracted the outer boundaries at the 0.5 isocontour level and converted them to polygons, discarding invalid or tiny shapes (area < 100). Last, all valid polygons were merged to yield a single tumor contour.

##### Ecotype identification

We defined “ecotypes” as recurrent spatial composition patterns of cellular states across Visium HD slides. To identify globally consistent ecotypes, we defined a two-stage pipeline: (i) within-sample discovery of local ecotypes from spatial neighborhoods, and (ii) cross-sample consensus meta-clustering of local ecotype profiles to obtain study-wide, global ecotypes.

1) Within-sample discovery of local ecotypes: For each Visium HD cell, we constructed a spatial *k*-nearest-neighbor (kNN) graph using Euclidean distance on the spatial coordinates (*k* = 50). Cell-type annotations were one-hot encoded, and for each cell, we averaged the one-hot vectors of its kNN to obtain a local composition vector representing the proportions of cell types in its immediate neighborhood. To account for the compositional nature of these vectors, we applied a centered log-ratio (CLR) transformation. Cells were then clustered on the CLR-transformed features using *K*-means (*n* = 6 clusters per sample). For interpretability and downstream consensus analysis, we computed a mean neighborhood composition profile for each local ecotype by averaging the composition vectors of its member cells.

2) Cross-sample consensus ecotypes: To generate global ecotypes, we performed agglomerative hierarchical clustering on all per-sample ecotype profiles. Rather than fixing the number of consensus clusters, we selected a distance threshold by maximizing the Calinski-Harabasz (CH) index over a grid of candidate cuts. Specifically, we computed pairwise cosine distances, formed a grid of 25 thresholds at the 10th to 90th percentiles of the empirical distance distribution, fit the hierarchy at each threshold, and retained the cut with the maximal CH on the original profile vectors. The selected threshold was used to obtain the final consensus ecotypes and their number *K*.

##### External Xenium analysis

To independently validate patterns of spatial compartmentalization, we analyzed high-resolution Xenium spatial transcriptomic data from (*14*). Tumor and nontumor cells were first distinguished directly in the Xenium dataset by scoring melanoma marker genes and classifying cells using a two-component Gaussian mixture model. Matched single-cell RNA-seq data from the same patients were annotated with tumor metaprograms and mapped to Xenium cells using the same OT framework described above, based on shared gene-expression profiles. Next, we computed the PLI for each metaprogram in the Xenium data. To validate our findings suggesting that R patients show higher levels of immune infiltration, we repeated the same OT-based procedure to annotate nontumor cells and quantified immune infiltration by considering the relative percentage of lymphocytes within the tumor region.

#### TE analyses

##### TE annotation

Repetitive DNA feature localizations (LINEs, SINEs, LTRs, and other categories such as DNA transposons and simple repeats) were retrieved from the UCSC RepeatMasker annotation (genome assembly GRCh38/hg38) using the AnnotationHub (v3.10.1) R package. The age of each TE instance was estimated based on sequence divergence, quantified as base mismatches per thousand (milliDiv) reported by RepeatMasker. Each milliDiv value was divided by the human genome substitution rate (2.2 × 10^−9^) to derive the final age (*92*).

#### TE matrix generation

For both snRNA-seq and snATAC-seq datasets, MATES (*60*) was used to estimate counts at the TE locus level from both unique and multimapping reads. Expression and accessibility matrices were subsequently generated by merging counts through summation, yielding a single matrix for each modality. Data were then normalized using size factors and log transformed. The resulting matrices were normalized using size factors, log transformed, and subsequently converted into Seurat object (v5.1.0) (*85*) for subsequent analysis and visualization.

##### Integration of modalities

TE-based RNA and ATAC modalities were integrated using the methods described above. PCs were selected based on the cellular subsets being analyzed. For the TME, RNA PCs 1 to 6 and ATAC PCs 1 to 10 were used. For malignant subsets, RNA PCs 1 to 9 and ATAC PCs 1 to 10 were considered for integration.

##### Differential expression and accessibility analyses

Differential expression and accessibility analyses between conditions were performed using the *scoreMarkers* function from the scran (v1.30.2) package. For each TE, rank-based statistics (*rank.AUC*, representing the relative ranking of the AUC across pairwise cluster comparisons, and *rank.logFC.cohen*, which ranks the standardized effect size or Cohen’s *d* of the log-fold change) were computed from both RNA and ATAC data to identify significant changes in expression and chromatin accessibility.

##### TE annotation on cCRE elements

TE loci overlapping MACS2-called peaks were further intersected with ENCODE cCREs and enhancer annotations from GeneHancer v4.4. cCRE-gene associations were retrieved from the ENCODE database to link TE-associated regulatory elements with their potential target genes.

##### TE-TAAs

A computational workflow was developed to identify TE-TAAs. This workflow consists of the following steps:

1) Tumor-expressed and accessible TEs. Differentially expressed and accessible TEs in malignant cells compared to immune cells were identified. The top 1% of TEs, ranked by logFC Cohen metric for both modalities (as described in the differential analysis section), were selected for downstream analysis.

2) Detection of potential peptide-coding TEs. Nucleotide sequences of each tumor-associated TE were translated in all six reading frames using a custom R function. Only putative open reading frames (ORFs) of at least 10 amino acids were retained.

3) Filtering of false-positive TE-derived peptides. Candidate peptides were aligned with BLASTp against methionine-initiated ORFs from the gEVE database (*93*). Only sequences with ≥95% identity and *e*-value <0.01 were retained as high-confidence TE-derived peptides.

4) MHC-I binding prediction. Putative TE-derived antigens were analyzed using netMHCpan and prioritized based on both MHC-I binding affinity and recognition potential scores, following the procedure described in (*10*)

5) High-confidence TE-TAAs. Last, an antigen quality metric integrating binding affinity and neoantigen recognition potential, which estimates the probability that a presented neoantigen will be recognized by the TCR repertoire (*94*), was applied to define high-confidence TE-TAAs.

A total of 154 unique TE-derived protein sequences were ultimately identified, originating from 84 distinct TE loci. From this pool, we predicted 161 potential antigenic peptides (8- to 11-mers) that passed all stringent filtering criteria and exhibited strong predicted binding affinity. To isolate minimal antigenic determinants, we collapsed overlapping sequences to generate a set of 99 unique core peptides. To confirm the physical presentation of these candidates, such a library was screened against published MS-based immunopeptidomic files obtained from (*63*).

To quantify the extent of malignant cells potentially undergoing immune elimination mediated by identified TE-TAAs, referred to as immunoedited cells, we calculated, for each MP, the percentage of cells that met the following criteria: (i) expression and chromatin accessibility at a TE locus giving rise to a TE-TAA (normalized counts >0), and (ii) identification of the corresponding TE-TAA as immunogenic in the matched patient sample. This percentage was computed relative to the total number of cells belonging to the same MP within each patient. Changes in such percentage were evaluated across treatment time points, considering week 0 as baseline and combining posttreatment samples collected at weeks 4 and 12.

#### Validation in independent melanoma cohorts

##### External bulk RNA-seq cohorts under ICI therapies

Bulk transcriptomic data and clinical information were obtained for eight independent cohorts of patients with melanoma treated with ICI. Six datasets (*4*, *95*–*99*) were acquired from the Tumor Immunotherapy Gene Expression Resource. Two additional datasets (*100*, *101*) were downloaded from cBioPortal and from the Gene Expression Omnibus (GEO) under accession number GSE168204, respectively. The composition of transcriptional metaprograms was inferred from the bulk RNA-seq expression matrix using the CIBERSORTx webserver (*102*). A custom signature matrix, generated from snRNA-seq data from this study, was used as the reference for deconvolution. The estimated tumor cell fractions were subsequently scaled based on tumor purity scores calculated with ESTIMATE (v1.0.13).

##### External scRNA-seq cohort under ICI therapy

A single-cell RNA-seq dataset of metastatic biopsies from patients with stage III and IV melanoma under ICI therapy (EGA accession EGAS00001006488) (*14*) was analyzed. To improve data quality, samples containing fewer malignant cells than the 10th percentile were excluded, and genes expressed in less than 1% of cells were removed. Expression of MP signatures was quantified using the *AddModuleScore* function, and cells were assigned to the signature with the highest score, retaining only assignments with a score greater than 0.05.

##### Internal snRNA-seq cohort under epi-immunotherapy

Two additional snRNA-seq samples from the NIBIT-M4 cohort, not included in the discovery phases of this study, were profiled as previously described. QC was performed in Seurat using sample-specific thresholds to account for differences in sequencing depth and complexity. Nuclei were excluded based on low or high gene complexity (<500 detected genes and >6500 or >4000 detected genes depending on the sample), elevated mitochondrial gene content (>5%), and abnormally high total transcript counts (nCount >30,000 or >10,000 depending on the sample). After QC, a total of 9570 nuclei were retained across the two samples. Malignant cells were annotated using SCEVAN (*16*), as previously described. MP signature expression was quantified using the *AddModuleScore* function, and metaprogram assignment was performed as described in the section “Malignant cell MP identification.”

#### Survival analysis

MP scores were analyzed as continuous variables (*z*-score standardized within each treatment group, per 1 SD increment). In parallel, samples were stratified into high- and low-MP groups according to the median MP composition calculated separately within each treatment group (pre- and post-ICI); the median-based cutoff was used to enable unbiased stratification and improve reproducibility across independent datasets. OS was computed in months from the date of treatment initiation. Survival analysis was performed using the Kaplan-Meier method, with group differences assessed by the log-rank test. Cox proportional hazards regression models were fitted to estimate HRs and corresponding 95% CIs, using both the continuous *z*-score MP scores and the median-derived categorical groups (high versus low). For each MP, four models were fitted: a univariate model and three multivariable models sequentially adjusted for (i) age and gender; (ii) age, gender, and disease stage; and (iii) disease stage and *BRAF* or *NRAS* mutational status. Multivariable models were fitted only when sample size and covariate completeness permitted convergence. HRs are reported on a log_2_ scale and visualized as forest plots. All statistical analyses were conducted in R using the survival package (v3.5-7).

#### Prognostic assessment

Prognostic value was evaluated in both the internal and external cohorts. For each sample, the relative proportions of metaprograms within the malignant compartment were calculated. Predictive performance for treatment response was estimated using leave-one-out cross-validation (LOOCV) with a generalized linear model implemented in caret (v6.0-94). Model accuracy was assessed by receiver operating characteristic (ROC) analysis, and the AUC was computed using pROC (v1.18.5).

#### In silico perturbation analysis

In silico perturbation of selected TFs was performed using the SCENIC+ workflow (*67*). For each TF, simulated perturbation outputs were generated across five independent iterations. Within each iteration, the expression values of genes belonging to the *Neural crest–like* metaprogram signature were averaged and compared against the baseline expression in cells assigned to this transcriptional state. The mean difference across signature genes was then computed per iteration, yielding an overall measure of the perturbation effect for each TF.

#### Gene regulatory network inference in the external scRNA-seq melanoma dataset

GRNs were inferred from the external scRNA-seq dataset (*14*), using pySCENIC (v0.12.1+8.gd2309fe) (*67*). To obtain a high-confidence network, the initial regulons were refined by applying a weight-based cutoff: For each TF, the 90th percentile of its target connection weights was used as a threshold, and only interactions exceeding this value were retained.

#### NFATC2 posttranscriptional activity

NFATC2 regulon activity was quantified on bulk RNA-seq datasets using the univariate linear model framework implemented in decoupleR (v2.9.7). The resulting activity score was subtracted from NFATC2 expression values to derive its posttranscriptional activity.

The predictive capacity of NFATC2 was assessed in pre- and post-ICI samples independently using LOOCV with a GLM as described above. Model performance was evaluated with ROC analysis and the corresponding AUC using pROC.

#### NFATC2 knockdown in melanoma cell lines

Gene expression data for NFATC2 knockdown and control melanoma cells were obtained from GEO (GSE101323) (*74*). Differential expression was performed with limma (v3.58.1). Gene set enrichment analysis was carried out using GO Biological Process, KEGG, Reactome, WikiPathways, and Hallmark collections (as described above). A single-sample MWW-GST (*84*) was applied using the top 50 up-regulated genes for each MP and the melanoma differentiation signatures from (*3*) to characterize the cell line.
