# Supplementary material for: Transposable elements and homotypic niches drive immune dynamics and resistance in melanoma epigenetic-based immunotherapy
Source: Sci Adv. 2026 Aug 7;12(32):eaed1318. doi: 10.1126/sciadv.aed1318 (PMC13450225; doi:10.1126/sciadv.aed1318)
Supplement: Supplementary file 1 [file sciadv.aed1318_sm.pdf]

Science Advances
AAAS

Supplementary Materials for

Transposable elements and homotypic niches drive immune dynamics and resistance in melanoma epigenetic-based immunotherapy

Erika Ciervo *et al.*

Corresponding author: Michele Maio, maio@unisi.it; Teresa Maria Rosaria Noviello, tmn58@med.miami.edu; Michele Ceccarelli, mxc2982@miami.edu

Sci. Adv. **12**, eaed1318 (2026)
DOI: 10.1126/sciadv.aed1318

The PDF file includes:

Figs. S1 to S9
Legends for tables S1 to S21

Other Supplementary Material for this manuscript includes the following:

Tables S1 to S21

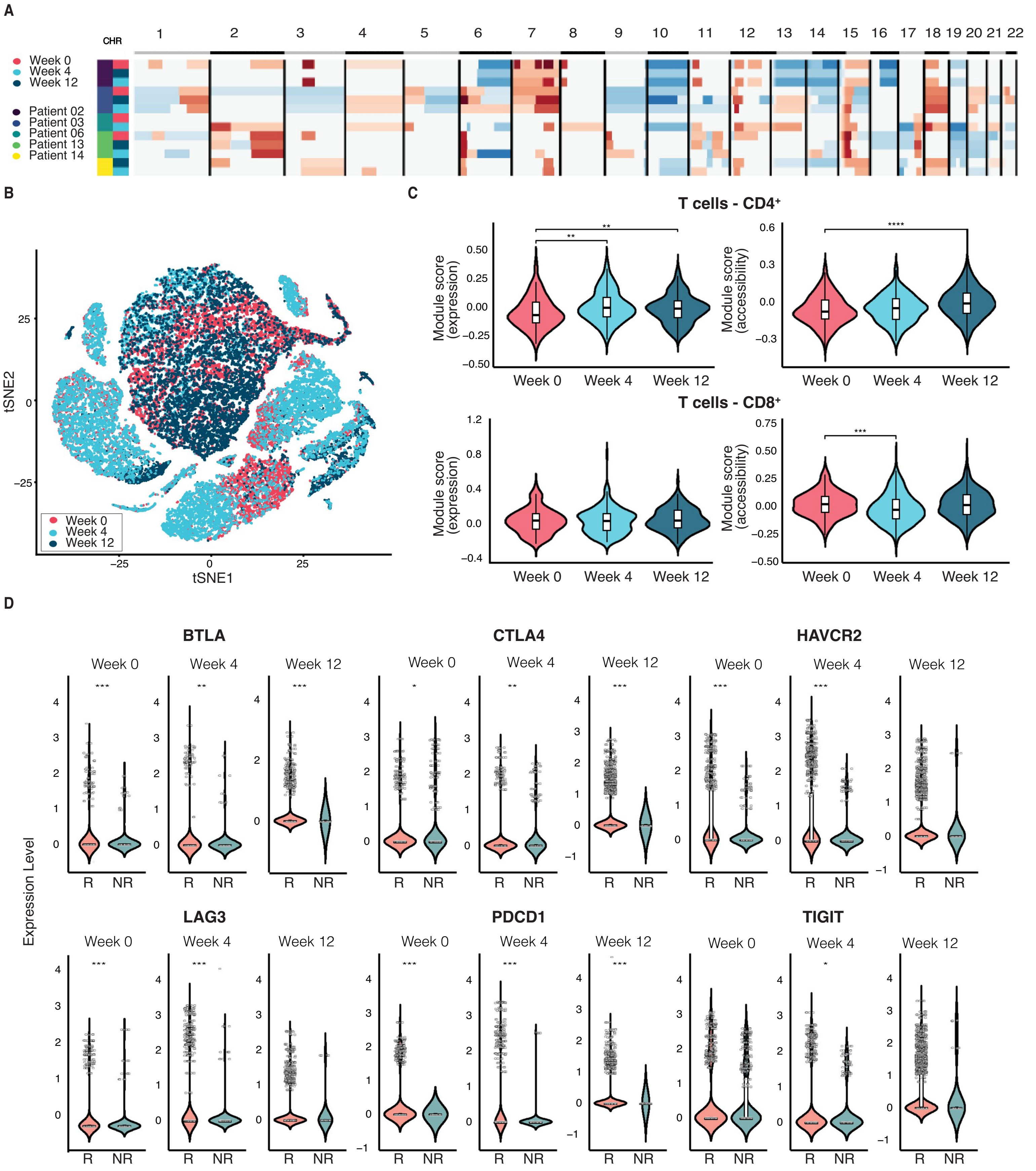

Fig. S1. Genomic and immune landscapes of the NIBIT-M4 cohort, related to Fig. 1.

A. Consensus heatmap of Copy Number Alterations (CNAs) in malignant cells inferred with SCEVAN *(16)*; patient IDs and sample time points are shown on the left track. CNAs are color-scaled from –2 (deep deletion, blue) to +2 (high amplification, red). **B.** t-SNE visualization of co-embedding of snRNA and snATAC modalities colored by time-points. **C.** Violin plots of Reactome PD1-signaling module scores from expression (left) and gene accessibility (right) in CD4⁺ (top) and CD8⁺ (bottom) T cells across time-points. Statistical significance was assessed using a two-sided Wilcoxon rank-sum test; ** $p \leq 0.01$, *** $p \leq 0.001$, **** $p \leq 0.0001$. **D.** Expression of immune checkpoint genes in R and NR patients across time-points. Statistical significance was assessed using a two-sided Wilcoxon rank-sum test; * $p \leq 0.05$, ** $p \leq 0.01$, *** $p \leq 0.001$.

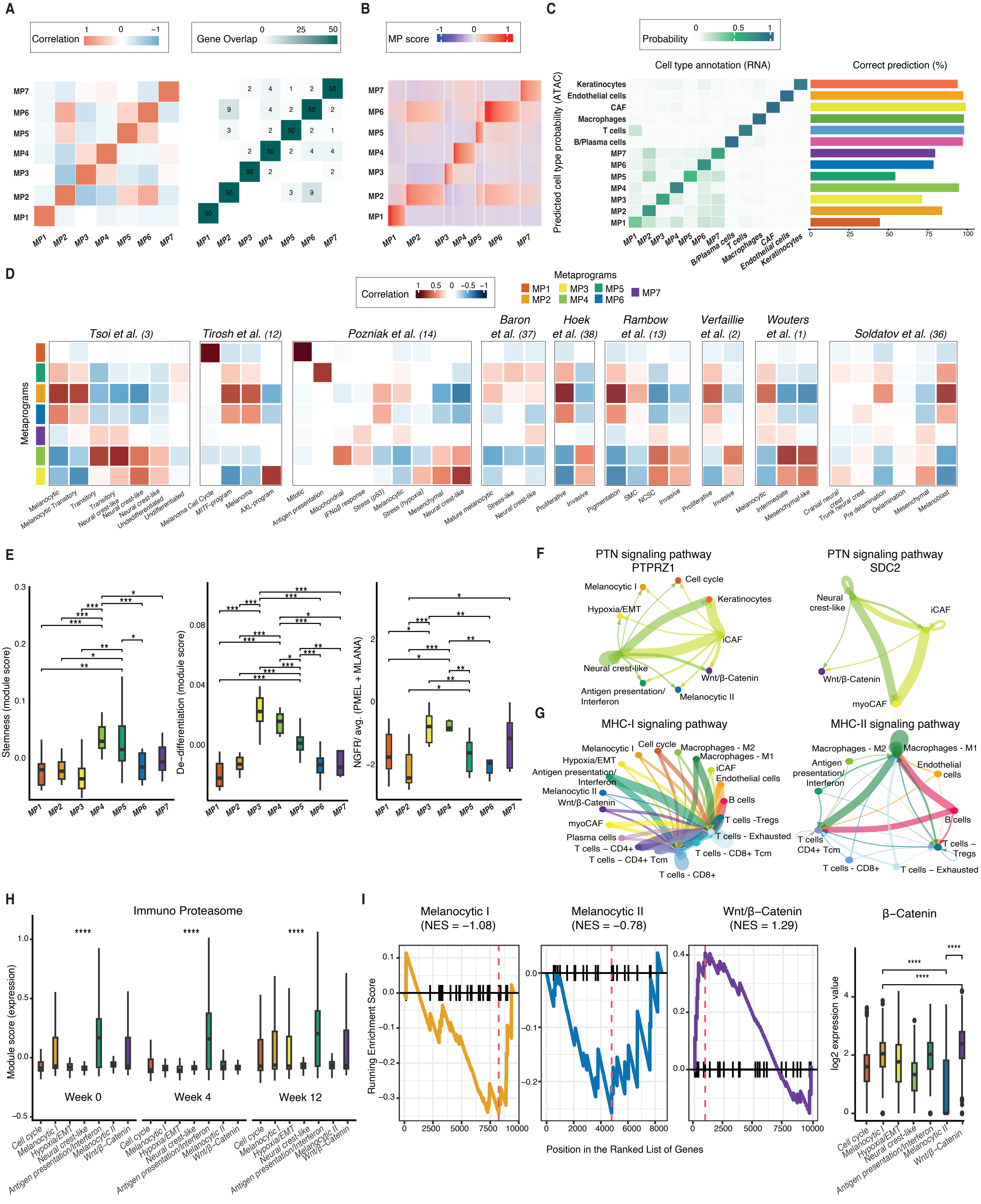
A
Correlation
1 0 −1
Gene Overlap
0 25 50
MP1 MP2 MP3 MP4 MP5 MP6 MP7
B
MP score
−1 0 1
C
Probability
0 0.5 1
Cell type annotation (RNA)
Correct prediction (%)
Predicted cell type probability (ATAC)
Keratinocytes
Endothelial cells
CAF
Macrophages
T cells
B/Plasma cells
0 25 50 75 100
D
Correlation
1 0.5 0 −0.5 −1
Metaprograms
MP1 MP2 MP3 MP4 MP5 MP6 MP7
Tsoi et al. (3)
Tirosh et al. (12)
Pozniak et al. (14)
Baron et al. (37)
Hoek et al. (38)
Rambow et al. (13)
Verfaillie et al. (2)
Wouters et al. (1)
Soldatov et al. (36)
Melanocytic
Melanocytic Transitory
Transitory
Transitory
Neural crest-like
Neural crest-like
Neural crest-like
Undifferentiated
Undifferentiated
Melanoma Cell Cycle
MITF-program
Melanoma
AXL-program
Mitotic
Antigen presentation
Mitochondrial
IFNαβ response
Stress (p53)
Melanocytic
Stress (hypoxia)
Mesenchymal
Neural crest-like
Mature melanocytic
Stress-like
Neural crest-like
Proliferative
Invasive
Pigmentation
SMC
NCSC
Invasive
Proliferative
Invasive
Melanocytic
Intermediate
Mesenchymal-like
Cranial neural crest
Trunk neural crest
Pre delamination
Delamination
Mesenchymal
Melanoblast
E
Stemness (module score)
De-differentiation (module score)
NGFR/ avg. (PMEL + MLANA)
F
PTN signaling pathway PTPRZ1
Cell cycle
Melanocytic I
Keratinocytes
Hypoxia/EMT
iCAF
Neural crest-like
Wnt/β-Catenin
Antigen presentation/ Interferon
Melanocytic II
PTN signaling pathway SDC2
Neural crest-like
iCAF
Wnt/β-Catenin
myoCAF
G
MHC-I signaling pathway
Melanocytic I
Cell cycle
Macrophages - M2
Macrophages - M1
Hypoxia/EMT
iCAF
Antigen presentation/ Interferon
Endothelial cells
Melanocytic II
Wnt/β-Catenin
B cells
myoCAF
T cells -Tregs
Plasma cells
T cells - Exhausted
T cells – CD4+
T cells - CD8+ Tcm
T cells – CD4+ Tcm
T cells - CD8+
MHC-II signaling pathway
Macrophages - M2
Macrophages - M1
Antigen presentation/ Interferon
Endothelial cells
B cells
T cells CD4+ Tcm
T cells – Tregs
T cells - CD8+
T cells – Exhausted
H
Immuno Proteasome
Module score (expression)
Week 0
Week 4
Week 12
Cell cycle
Melanocytic I
Hypoxia/EMT
Neural crest-like
Antigen presentation/Interferon
Melanocytic II
Wnt/β-Catenin
I
Melanocytic I (NES = −1.08)
Melanocytic II (NES = −0.78)
Wnt/β−Catenin (NES = 1.29)
Running Enrichment Score
Position in the Ranked List of Genes
β−Catenin
log2 expression value

fig. S2 Functional dissection of metaprograms, related to Fig. 2.

A. Heatmaps showing Pearson correlations between each MP score and all other MP scores (left), and 50-NMF gene overlap (right) between MPs. **B.** Heatmap of MP scores across cells. Rows represent MPs, columns represent cells. Cells were aggregated by MPs and ordered according to their scores. **C.** Heatmap of average percentage score of cells from a given expression-defined state (columns) assigned to different cell states (rows) based on gene accessibility profiles, with barplot of prediction accuracy. **D.** Heatmaps showing Pearson correlations between MP scores with previously published melanoma signatures *(1–3, 12–14 , 36–38)*. **E.** Boxplots showing the distribution of stemness module scores (*40*) (left), de-differentiation signature (*41*) (middle) and the ratio between NGFR expression over the average expression of *PMEL* and *MLANA* (*42*) (right) in tumor cells classified into distinct transcriptional MPs. Statistical significance between groups was assessed using a two-sided Wilcoxon rank-sum test; * $p \leq 0.05$, ** $p \leq 0.01$, *** $p \leq 0.001$. **F.** CellChat aggregated networks of selected pathways in NR patients; line width indicates interaction probability. **G.** Same analysis as in (**F**), shown for R patients. **H.** Boxplots of immunoproteasome (*PSMB8*, *PSMB9*, *PSMB10*) module scores across MPs at different time-points. Statistical significance was assessed using Kruskal-Wallis test; **** $p \leq 0.0001$. **I.** Wnt/β-catenin program characterization with enrichment plots of HALLMARK_WNT_BETA_CATENIN_SIGNALING pathway in *Melanocytic I*, *Melanocytic II* and *Wnt/β-catenin* MPs, and β-catenin gene expression ($\log_2$ normalized counts). Statistical significance was assessed using a two-sided Wilcoxon rank-sum test; **** $p \leq 0.0001$.

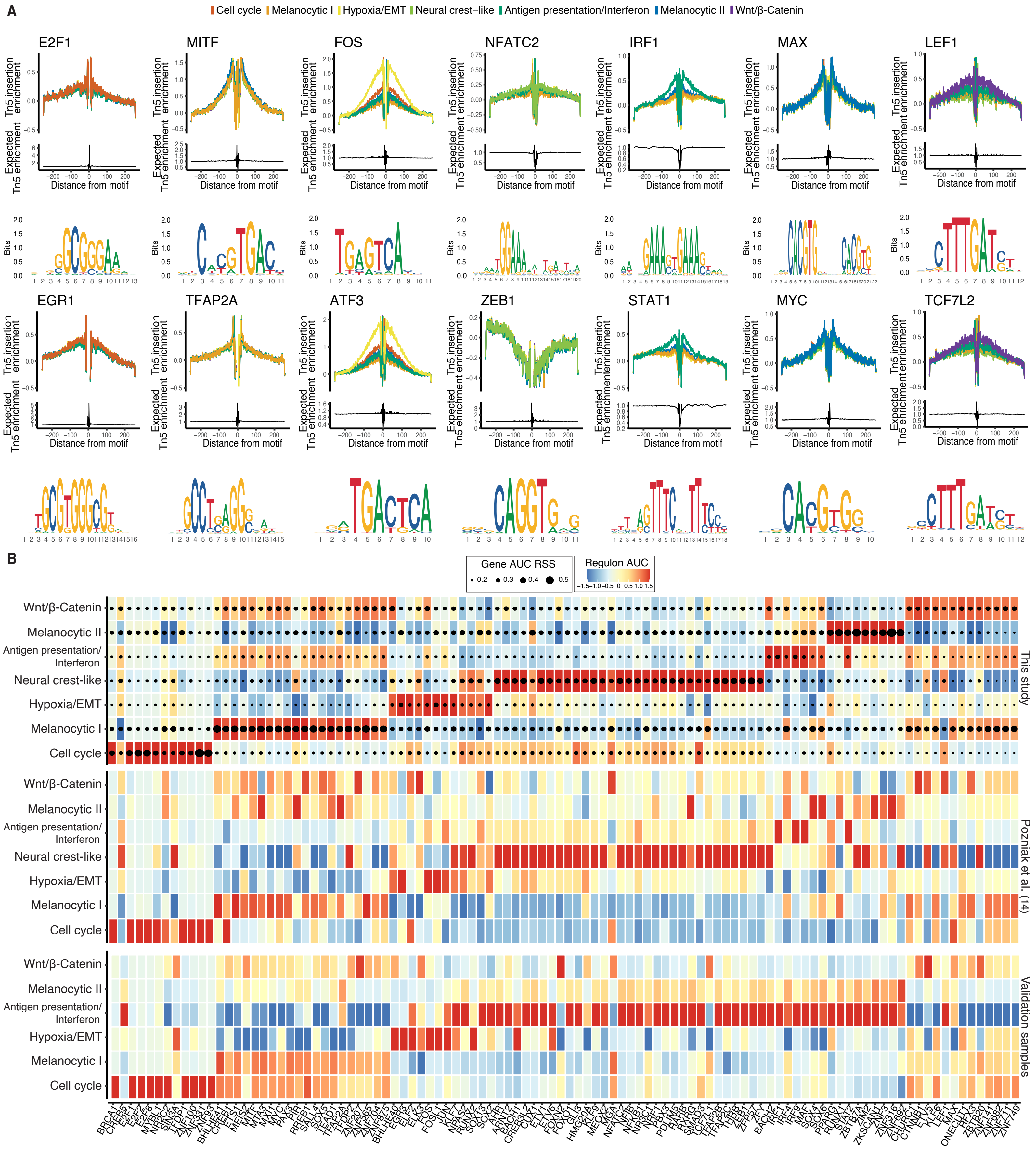

fig. S3 Transcription factor activity across metaprograms, related to Fig. 2.

A. Footprinting analysis of representative TFs per MP showing chromatin accessibility patterns (top) together with sequence logos of their binding motifs (bottom). **B.** TF eRegulon profiles across MPs. Top: heatmap/dot plot displaying TF expression (AUCell score, color scale) and eRegulon cell-type specificity measured by the regulon specificity score (RSS; point size) across MPs identified in this study. Middle: heatmap displaying TF expression (AUCell score, color scale) across MPs from Pozniak et al. *(14)*, after assigning each cell to the identified MPs. Bottom: heatmap displaying TF expression (AUCell score, color scale) across MPs from two additional validation samples from this study. Neural crest-like cells were not detected in these samples.

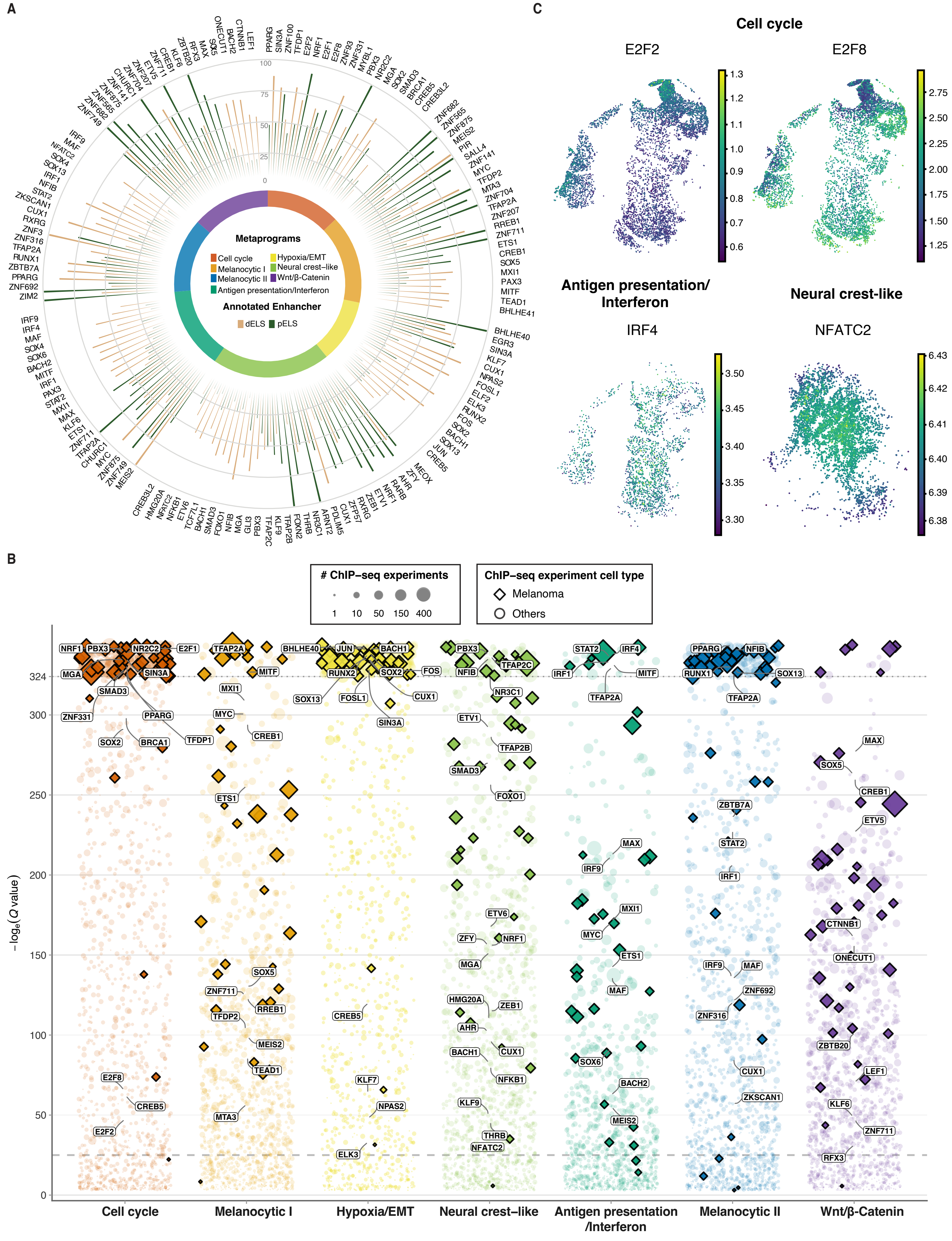
A
Metaprograms
Cell cycle
Melanocytic I
Melanocytic II
Antigen presentation/Interferon
Hypoxia/EMT
Neural crest-like
Wnt/β-Catenin
Annotated Enhancher
dELS
pELS
0
25
50
75
100
C
Cell cycle
E2F2
E2F8
Antigen presentation/ Interferon
Neural crest-like
IRF4
NFATC2
B
ChIP−seq experiments
1
10
50
150
400
ChIP−seq experiment cell type
Melanoma
Others
−log_e(Q value)
0
50
100
150
200
250
300
324
Cell cycle
Melanocytic I
Hypoxia/EMT
Neural crest−like
Antigen presentation /Interferon
Melanocytic II
Wnt/β-Catenin

fig. S4 Transcription factor binding and spatial activity across the metaprograms.

A. Circular plot showing the percentage of SCENIC+ -predicted TF binding regions overlapping ENCODE-annotated proximal (pELS) and distal (dELS) enhancer-like elements across the seven metaprograms. **B.** ChIP-seq–based validation of metaprogram-specific transcription factor binding. The bubble plot shows the enrichment of TF binding at metaprogram-specific accessible chromatin peaks, assessed using ChIP-Atlas *(87)*. Enrichment was assessed by Fisher's exact test with Benjamini–Hochberg multiple testing correction; only features with $Q < 0.05$ were retained. The y-axis displays the −log(Q-value), TFs identified by SCENIC+ regulon inference are highlighted. **C.** Spatial transcriptomic maps showing the expression of representative TFs predicted to drive distinct metaprograms. Top: Expression of *E2F2* and *E2F8* is enriched in proliferative *(Cell cycle)* regions. Bottom left: *IRF4* localizes to *Antigen presentation/Interferon* associated areas. Bottom right: *NFATC2* is preferentially expressed in *Neural crest–like* regions.

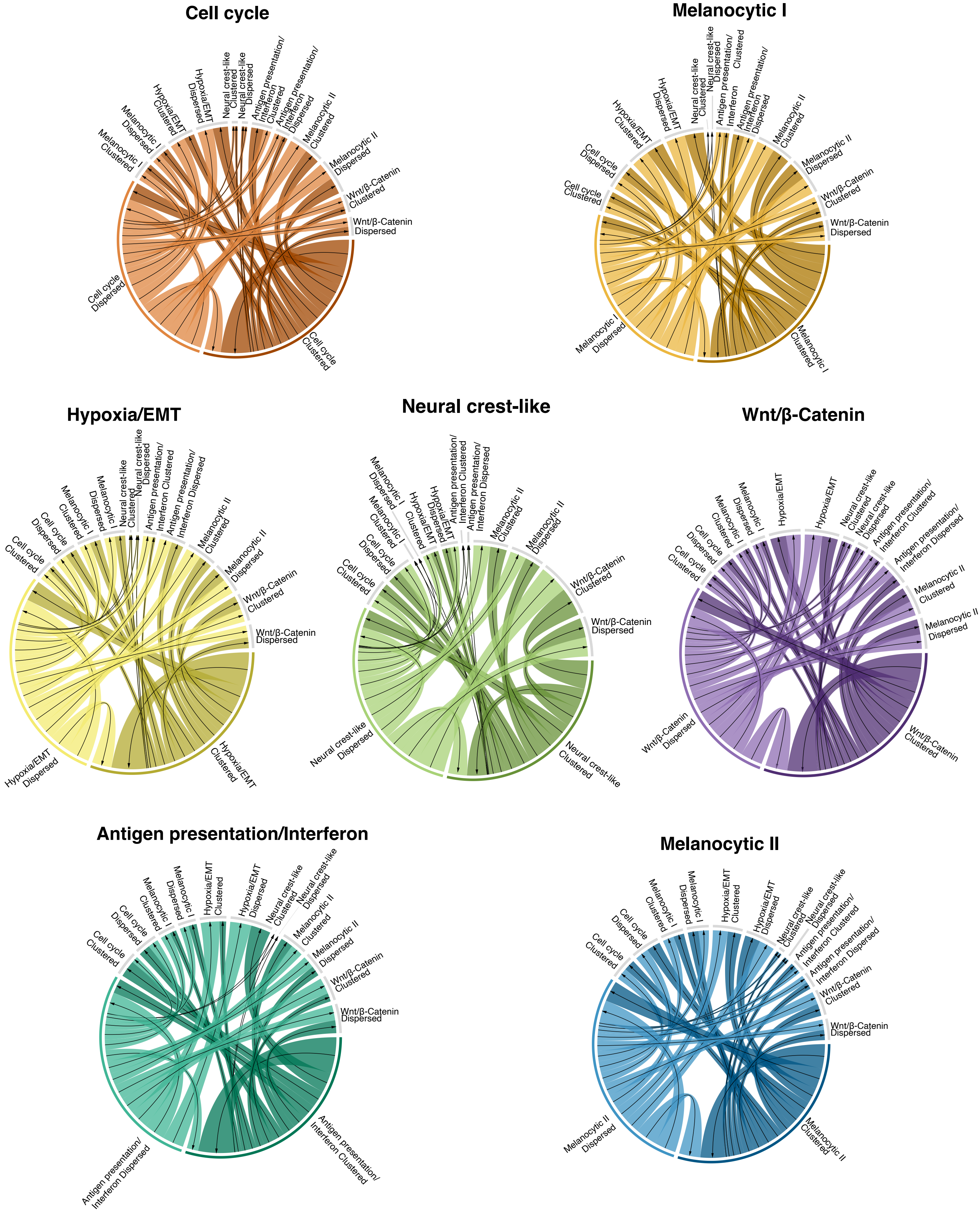

fig. S5 Transition probabilities for the malignant cell states conditioned on their spatial configuration.
For all MPs, the clustered cells showed a higher probability to transition into the same state and retain their spatial organization compared to the dispersed states. The strongest homotypic transitions were observed in the *Antigen presentation/Interferon* and *Hypoxia/EMT* states.

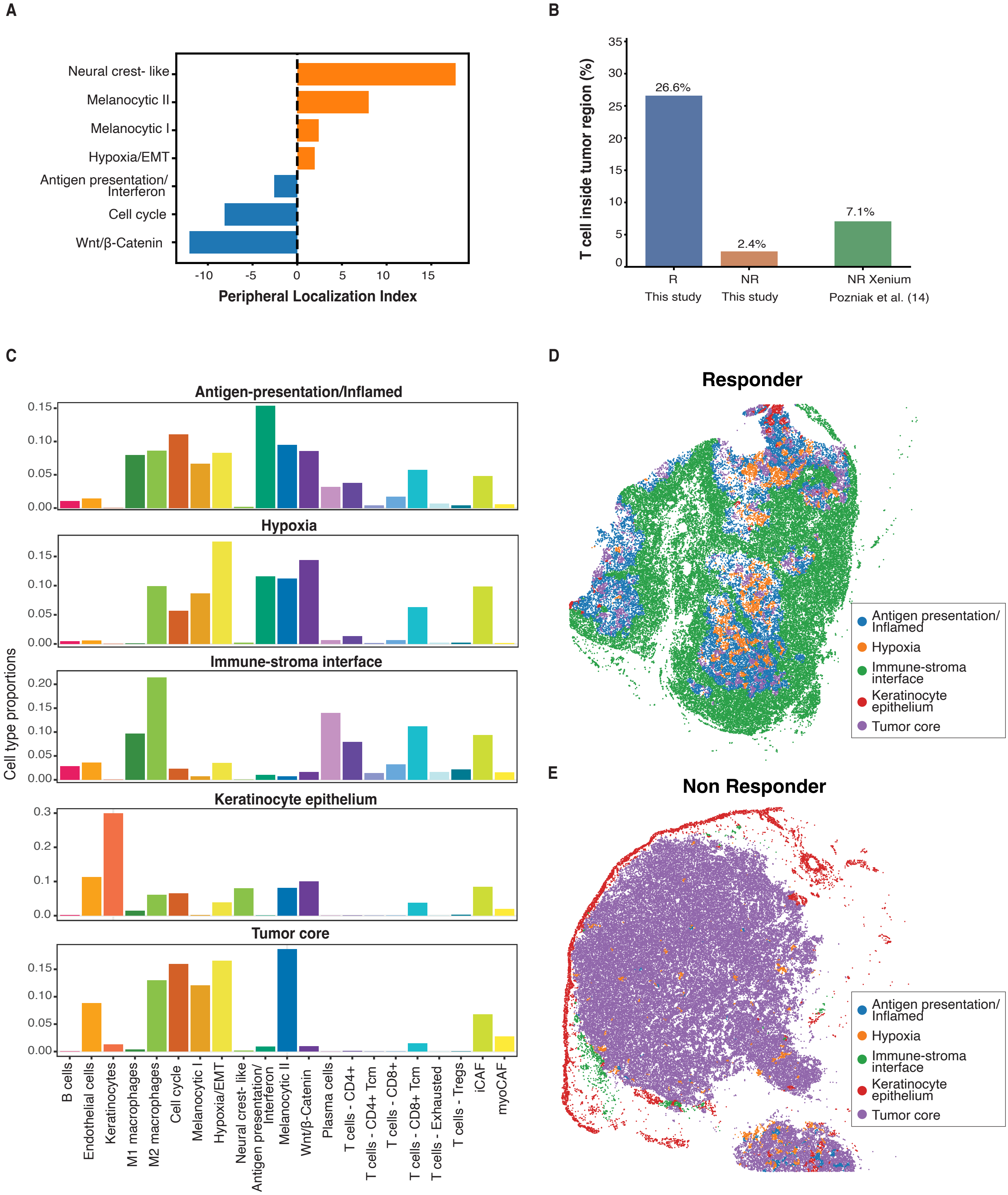

fig. S6 External validation on Xenium data and unsupervised ecotypes from Visium HD samples.

A. Peripheral Localization Index on high-resolution Xenium data from Pozniak et al. *(14)*. **B.** Proportion of lymphocytes within the tumor region for R and NR Visium HD samples and NR Xenium samples. **C.** Proportions of cell types in each ecotype. The ecotype composition was used to label them as Antigen-presentation/Inflamed, Hypoxia, Immune–stroma interface, Keratinocyte epithelium, and Tumor core. **D.** Spatial distribution of the identified ecotypes in a responder and non-responders sample **E.**, respectively.

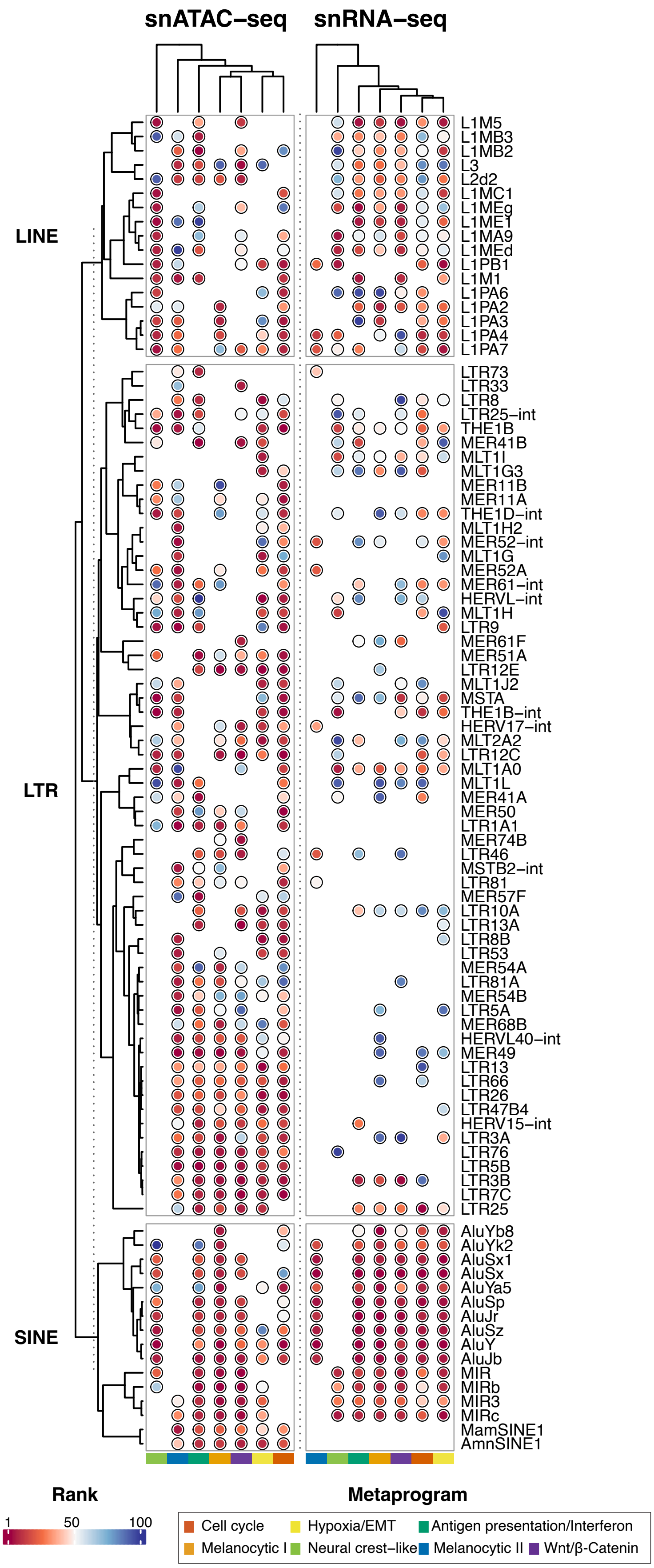

fig. S7 Differentially active TEs in tumor metaprograms.
Dot heatmaps show the top-ranked TEs based on *rank.AUC*, reflecting the relative ranking of the Area Under the Curve (AUC) across pairwise cluster comparisons, for both scATAC-seq (left) and scRNA-seq (right). TEs with rank values approaching 1 (indicated in red) exhibit the strongest discriminatory power for specific cell populations. TE class annotations are provided on the left, and the metaprogram color track is displayed below.

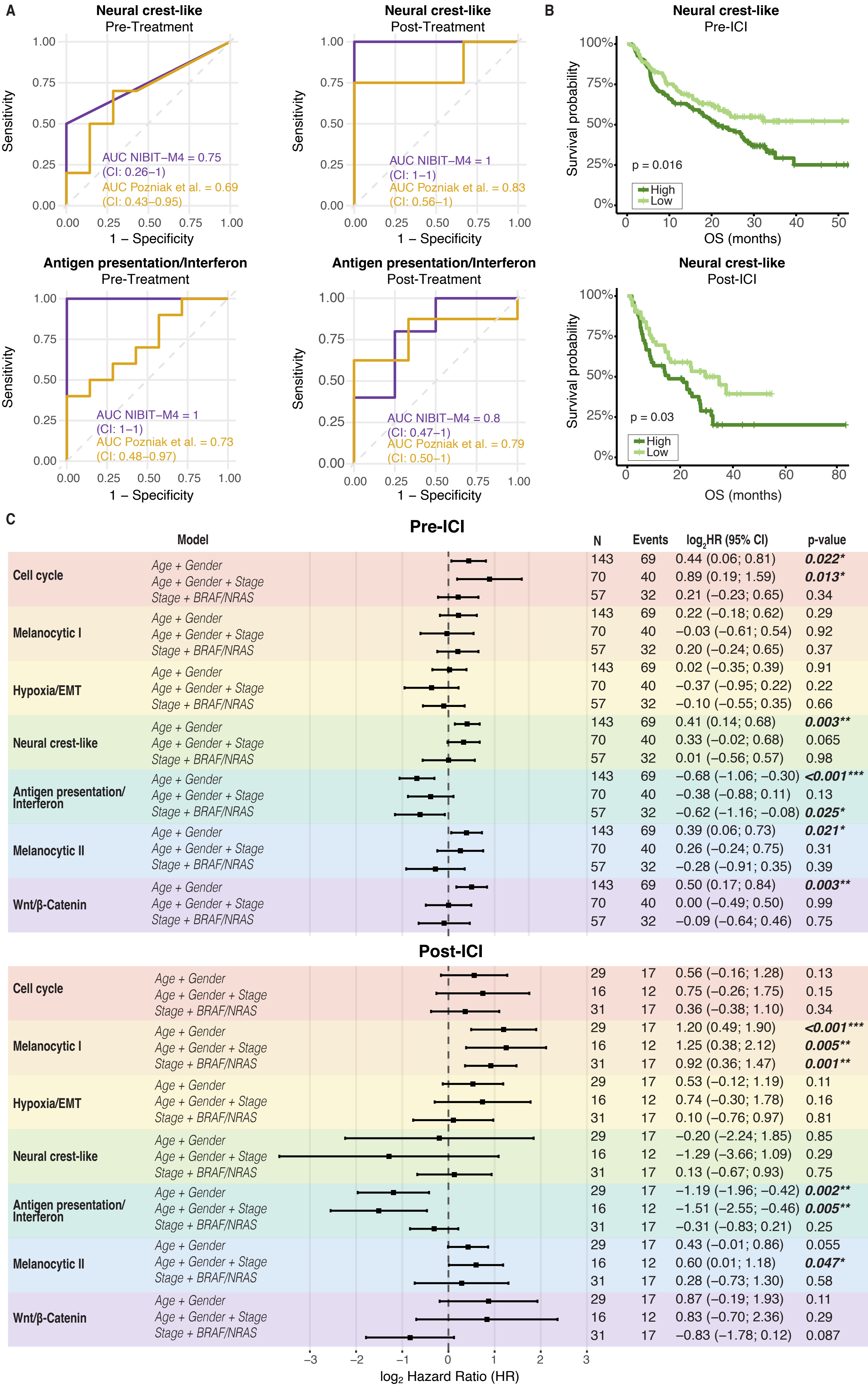
A
Neural crest-like
Pre-Treatment
AUC NIBIT–M4 = 0.75
(CI: 0.26–1)
AUC Pozniak et al. = 0.69
(CI: 0.43–0.95)
Neural crest-like
Post-Treatment
AUC NIBIT–M4 = 1
(CI: 1–1)
AUC Pozniak et al. = 0.83
(CI: 0.56–1)
Antigen presentation/Interferon
Pre-Treatment
AUC NIBIT–M4 = 1
(CI: 1–1)
AUC Pozniak et al. = 0.73
(CI: 0.48–0.97)
Antigen presentation/Interferon
Post-Treatment
AUC NIBIT–M4 = 0.8
(CI: 0.47–1)
AUC Pozniak et al. = 0.79
(CI: 0.50–1)
Sensitivity
1 – Specificity
B
Neural crest-like
Pre-ICI
p = 0.016
Neural crest-like
Post-ICI
p = 0.03
High
Low
Survival probability
OS (months)
C
Pre-ICI
Model
N
Events
log2HR (95% CI)
p-value
Cell cycle
Age + Gender 143 69 0.44 (0.06; 0.81) 0.022*
Age + Gender + Stage 70 40 0.89 (0.19; 1.59) 0.013*
Stage + BRAF/NRAS 57 32 0.21 (−0.23; 0.65) 0.34
Melanocytic I
Age + Gender 143 69 0.22 (−0.18; 0.62) 0.29
Age + Gender + Stage 70 40 −0.03 (−0.61; 0.54) 0.92
Stage + BRAF/NRAS 57 32 0.20 (−0.24; 0.65) 0.37
Hypoxia/EMT
Age + Gender 143 69 0.02 (−0.35; 0.39) 0.91
Age + Gender + Stage 70 40 −0.37 (−0.95; 0.22) 0.22
Stage + BRAF/NRAS 57 32 −0.10 (−0.55; 0.35) 0.66
Neural crest-like
Age + Gender 143 69 0.41 (0.14; 0.68) 0.003**
Age + Gender + Stage 70 40 0.33 (−0.02; 0.68) 0.065
Stage + BRAF/NRAS 57 32 0.01 (−0.56; 0.57) 0.98
Antigen presentation/ Interferon
Age + Gender 143 69 −0.68 (−1.06; −0.30) <0.001***
Age + Gender + Stage 70 40 −0.38 (−0.88; 0.11) 0.13
Stage + BRAF/NRAS 57 32 −0.62 (−1.16; −0.08) 0.025*
Melanocytic II
Age + Gender 143 69 0.39 (0.06; 0.73) 0.021*
Age + Gender + Stage 70 40 0.26 (−0.24; 0.75) 0.31
Stage + BRAF/NRAS 57 32 −0.28 (−0.91; 0.35) 0.39
Wnt/β-Catenin
Age + Gender 143 69 0.50 (0.17; 0.84) 0.003**
Age + Gender + Stage 70 40 0.00 (−0.49; 0.50) 0.99
Stage + BRAF/NRAS 57 32 −0.09 (−0.64; 0.46) 0.75
Post-ICI
Cell cycle
Age + Gender 29 17 0.56 (−0.16; 1.28) 0.13
Age + Gender + Stage 16 12 0.75 (−0.26; 1.75) 0.15
Stage + BRAF/NRAS 31 17 0.36 (−0.38; 1.10) 0.34
Melanocytic I
Age + Gender 29 17 1.20 (0.49; 1.90) <0.001***
Age + Gender + Stage 16 12 1.25 (0.38; 2.12) 0.005**
Stage + BRAF/NRAS 31 17 0.92 (0.36; 1.47) 0.001**
Hypoxia/EMT
Age + Gender 29 17 0.53 (−0.12; 1.19) 0.11
Age + Gender + Stage 16 12 0.74 (−0.30; 1.78) 0.16
Stage + BRAF/NRAS 31 17 0.10 (−0.76; 0.97) 0.81
Neural crest-like
Age + Gender 29 17 −0.20 (−2.24; 1.85) 0.85
Age + Gender + Stage 16 12 −1.29 (−3.66; 1.09) 0.29
Stage + BRAF/NRAS 31 17 0.13 (−0.67; 0.93) 0.75
Antigen presentation/ Interferon
Age + Gender 29 17 −1.19 (−1.96; −0.42) 0.002**
Age + Gender + Stage 16 12 −1.51 (−2.55; −0.46) 0.005**
Stage + BRAF/NRAS 31 17 −0.31 (−0.83; 0.21) 0.25
Melanocytic II
Age + Gender 29 17 0.43 (−0.01; 0.86) 0.055
Age + Gender + Stage 16 12 0.60 (0.01; 1.18) 0.047*
Stage + BRAF/NRAS 31 17 0.28 (−0.73; 1.30) 0.58
Wnt/β-Catenin
Age + Gender 29 17 0.87 (−0.19; 1.93) 0.11
Age + Gender + Stage 16 12 0.83 (−0.70; 2.36) 0.29
Stage + BRAF/NRAS 31 17 −0.83 (−1.78; 0.12) 0.087
log2 Hazard Ratio (HR)

fig. S8 Prognostic significance of metaprograms and NFATC2 functional regulation, related to Fig. 6.

A. Receiver operating characteristic (ROC) curves of response prediction for the fraction of *Neural crest-like* (top) and *Antigen presentation/Interferon* (bottom) cells among malignant cells in the NIBIT-M4 cohort (violet) and external cohort (*14*) (goldenrod), shown pre-treatment (left) and post-treatment (right). Areas under the ROC curve (AUCs) are reported with 95% confidence intervals (CIs). **B.** Kaplan–Meier curves of overall survival (OS) in bulk melanoma cohorts, shown separately for pre-ICI (left) and post-ICI (right). Patients were divided into High (dark green) and Low (light green) groups based on the median fraction of *Neural crest-like* cells. Statistical significance was assessed using the log-rank test. **C.** Forest plots of Cox regression models for overall survival in pre-ICI (top) and post-ICI (bottom) cohorts. Each metaprogram score was z-scored and tested as a continuous variable (per 1 SD increment). Three multivariable models are shown per MP, adjusted for age and gender; age, gender and disease stage; or disease stage and *BRAF* or *NRAS* mutational status. Hazard ratios (HR) are shown on a $\log_2$ scale with 95% confidence intervals (CIs); *$p < 0.05$, **$p < 0.01$, ***$p < 0.001$.

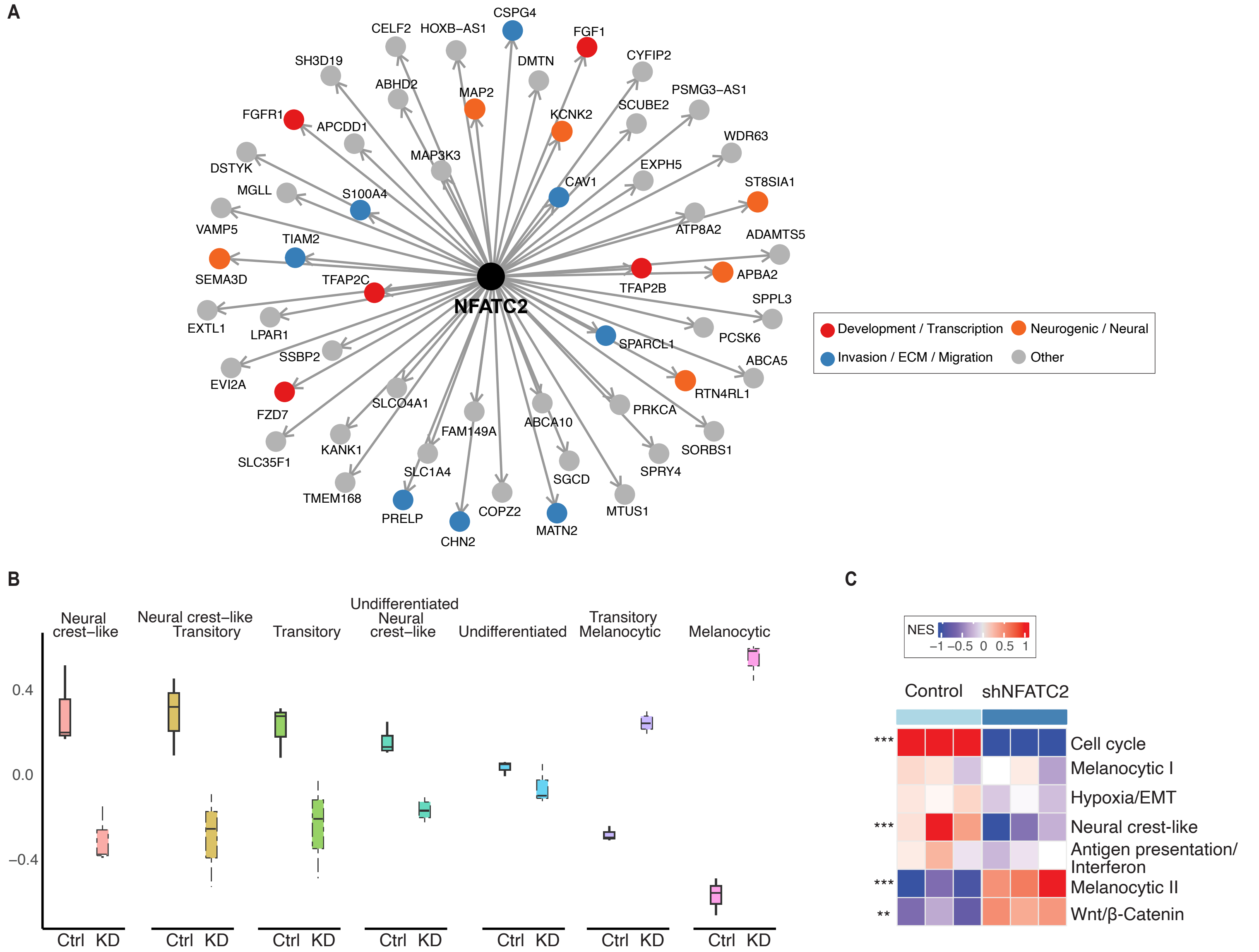

fig. S9 NFATC2 regulates melanoma differentiation, related to Fig.6.

A. NFATC2 regulon network showing gene targets as nodes, colored by major functional category. **B.** Boxplots showing the Normalized Enrichment Scores (NES) of melanoma differentiation signatures *(3)* under control (Ctrl) and knockdown (KD) conditions. **C.** Heatmap of single-sample NES for the top 50 DEGs per MP in control and shRNA *NFATC2* conditions. NES values are displayed on a –1 to +1 color scale (blue to red). Statistical significance was calculated using combined p-values across replicates with Fisher's method; ** $p \leq 0.01$, *** $p \leq 0.001$.

Other supplementary materials: Table S1 to S21 as excel files

- **Table S1.** Correlation between CNV profiles between snRNA-seq and bulk WES from patient samples with matched normal blood available from Noviello et al., 2023 *(10).*
- **Table S2.** List of upregulated genes (avg_log2FC > 1.5 & p_adjust < 0.05) in each cell type.
- **Table S3.** List of top 50 NMF-derived genes for each metaprogram.
- **Table S4.** Enrichment results (over-representation test) on curated gene sets using the top 50 NMF-derived genes for each metaprogram.
- **Table S5.** DEGs comparing each metaprogram vs. all others.
- **Table S6.** GSEA results on curated gene sets by metaprogram comparison.
- **Table S7.** List of top 30 enriched transcription factor motifs for each metaprogram.
- **Table S8.** List of prioritized transcription factors obtained with SCENIC+.
- **Table S9.** Percentage of SCENIC+-predicted TF binding regions overlapping ENCODE-annotated proximal (pELS) and distal (dELS) enhancer-like elements.
- **Table S10.** Percentage of SCENIC+-predicted TF binding regions overlapping ENCODE-annotated proximal (pELS) and distal (dELS) enhancer-like elements across 49 annotated tissues.
- **Table S11.** List of enrichment results on curated gene sets using MP-specific target genes of enriched transcription factors across cCREs
- **Table S12.** ChIP-seq enrichment analysis results for each metaprogram.
- **Table S13.** Tumor cells profiled by Xenium (from Pozniak et al., 2024 *(14)*), annotated with Optimal Transport -derived metaprogram (MP) identities.
- **Table S14.** Differential analysis of tumor microenvironment (TME) cells between baseline (week 0, before therapy) and on-treatment (week 4), reporting for each transposable element (TE) comparative ATAC and RNA metrics.
- **Table S15.** Differential analysis of malignant cells for each metaprogram versus all others, reporting for each transposable element (TE) comparative ATAC and RNA metrics.
- **Table S16.** Predicted TE-derived antigens, including their repetitive element (RE) origin, HLA-binding affinity, genomic localization within open reading frames (ORFs), and their corresponding validation status in mass spectrometry-based immunopeptidomics datasets.
- **Table S17.** TE-TAAs present in independent mass spectrometry (MS)-based immunopeptidomics datasets.
- **Table S18.** Clinical data and metaprogram fraction deconvolution results from the bulk-RNA ICI dataset.
- **Table S19.** Hazard ratios (HR), 95% confidence intervals, and p-values from univariate and multivariable Cox regression models for each metaprogram score (continuous or categorical) in bulk-RNA ICI dataset.
- **Table S20.** DEGs comparing NFATC2-KD samples vs. control.
- **Table S21.** GSEA results on Gene Ontology Biological Process gene sets from the NFATC2-KD comparison.
